# Meta-analysis of human methylomes reveals stably methylated sequences surrounding CpG islands associated with high gene expression

**DOI:** 10.1186/1756-8935-7-28

**Published:** 2014-10-23

**Authors:** Rachel Edgar, Powell Patrick Cheng Tan, Elodie Portales-Casamar, Paul Pavlidis

**Affiliations:** Genome Science and Technology Graduate Program, University of British Columbia, 2329 W Mall, Vancouver, BC V6T 1Z4 Canada; Centre for High-Throughput Biology and Department of Psychiatry, University of British Columbia, 2890 E Mall, Vancouver, BC V6T 1Z4 Canada

## Abstract

**Background:**

DNA methylation is thought to play an important role in the regulation of mammalian gene expression, partly based on the observation that a lack of CpG island methylation in gene promoters is associated with high transcriptional activity. However, the CpG island methylation level only accounts for a fraction of the variance in gene expression, and methylation in other domains is hypothesized to play a role. We hypothesized that regions of very high stability in methylation would exist and provide biological insight into the role of methylation both within and outside CpG islands.

**Results:**

We set out to identify highly stable regions in the human methylome, based on the subset of CpGs assayed with an Illumina Infinium 450 K array. Using 1,737 samples from 30 publically available studies, we identified 15,224 CpGs that are ‘ultrastable’ in their state across tissues and developmental stages (974 always methylated; 14,250 always unmethylated). Further analysis of ultrastable CpGs led us to identify a novel subset of CpG islands, ‘ravines’, which exhibit a markedly consistent pattern of low methylation with highly methylated flanking shores and shelves. We distinguish ravines from other CpG islands characterized by a broader flanking region of low methylation. Interestingly, ravines are associated with higher gene expression compared to typical unmethylated CpG islands, and are more often found near housekeeping genes.

**Conclusions:**

The identification of ultrastable sites in the human methylome led us to identify a subclass of CpG islands characterized by a very stable pattern of methylation encompassing the island and flanking regions, established early in development and maintained through differentiation. This pattern is associated with particularly high levels of gene expression, providing new evidence that methylation beyond the CpG island could play a role in gene expression.

**Electronic supplementary material:**

The online version of this article (doi:10.1186/1756-8935-7-28) contains supplementary material, which is available to authorized users.

## Background

Variation in the methylation state of DNA across cell types, developmental stages and physiological or disease conditions is of intense interest to understanding mammalian gene regulation. To this end, numerous studies have been carried out to measure DNA methylation states among cell types or conditions at the resolution of single cytosine guanine dinucleotides (CpGs). Currently, the field is undergoing an explosion of characterization of methylomes, leading to a growing but still highly incomplete understanding of the relationships among methylation, gene expression, normal cellular function and disease [1]. The conceptually simplest approach is to divide chromosomes into domains or clusters of similar methylation states and correlate such domains with the location of genes or their regulatory sequences, and with other epigenetic marks such as histone acetylation or methylation. However, even with massive efforts such as ENCODE [2], numerous gaps in our knowledge exist, particularly in the variation (and functional significance) of epigenetic states across multiple cell types and conditions.

Early studies focused on CpG islands (CGIs), defined as short (approximately 1 kb) regions of high CpG density in an otherwise CpG-sparse genome [3]. Many CGIs are associated with gene promoters [4, 5], and methylation at CGIs is associated with repression of transcription [6, 7]. More recently, the utility of the concept of the CGI has been challenged as it has become more technologically feasible to directly measure methylation, rather than relying on inferred states based on CpG density [8]. Genome-wide analysis has thus helped define a growing geography of biologically significant methylation patterns besides that associated with CGIs near promoters. CGI ‘shores’, defined as the 2 kb of sequence flanking a CGI, have been reported to be more dynamic than the CGI itself [9, 10]. Beyond shores are ‘shelves’ [11] and ‘open sea’ sites [12]. More recently, large DNA methylation ‘valleys’ and ‘canyons’ of low methylation have been identified [13–15]. Other domains, identified in tumor cells, are termed ‘low-methylated regions’ (LMRs) and ‘long-range epigenetic activation’ (LREA) or silencing (LRES) domains of relatively low or high methylation [16–18]. We note that the definition of these domains inevitably relies on investigator-specified parameters of length and methylation level, and they are not mutually exclusive; for example, canyons often overlap CGIs. In addition, the relative stability of domains such as LREAs and canyons across cell types and conditions is still not completely documented.

In general, the largest changes in DNA methylation are seen during development, which involves global methylation erasure and reestablishment [19], and in cancer, which is characterized by extensive and often gene-specific changes compared to normal tissues [20]. Beyond this, many studies have emphasized the general stability of the methylome. Even between different tissues or tumor types, the number of differentially methylated CpGs reported ranges from 0.5% to 20% (depending in part on the statistical tests and significance cut-offs; [21, 22]). Understanding which sites and domains are relatively static or dynamic is an important step to assigning function to DNA methylation.

Because many previous studies focused on differences in methylation across conditions or cell types, there is likely to be additional information on stability waiting to be identified. Here we analyze a large collection of DNA methylation data to identify a set of ultrastable CpG sites. We associate many of these sites with a novel subset of CGIs we refer to as ‘ravines’, which tend to be near housekeeping genes and associated with high expression activity and open chromatin states. We propose a new classification of CGIs that takes into account the methylation state of the island as well as the shores and shelves.

## Results

### Ultrastable DNA methylation sites

**Figure 1:**
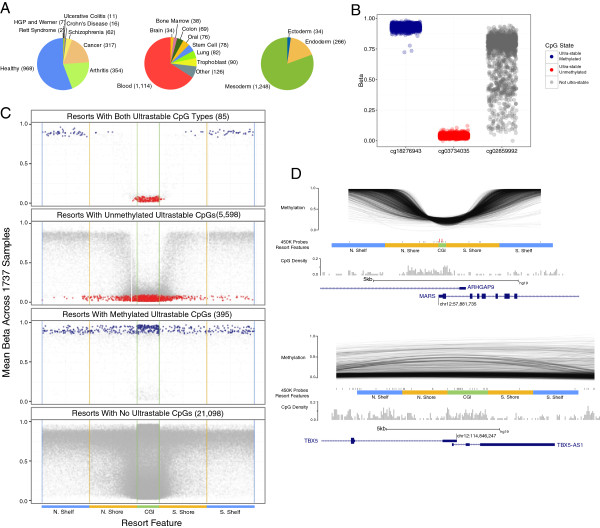
**Ultrastable cytosine guanine dinucleotides** **(CpGs) highlight a novel class of CpG islands (CGI). (** **A** **)** Counts of 450 K samples disease tissue and germ layer samples used in analysis. [See Additional file 1: Table S2 for complete list of tissue types used]. **(** **B** **)** Representative CpGs of the methylation stability states (not ultrastable, ultrastable unmethylated and ultrastable methylated). Points represent an individual sample. Color scheme for ultrastable CpGs is maintained throughout the paper **(** **C** **)** Ultrastable CpGs allow observation of a unique resort methylation pattern. Composite profiles are shown for all 27,176 resorts on the 450 K. As CGIs have variable lengths, the CpG position within a CGI is shown here as relative to the length of the CGI. CGIs are plotted as 935.23 bp (mean length of all CGIs measured on the 450 K). Beyond the CGI boundaries on the plot (that is, start at 0 and end at 935.23), the CpGs actual distance, in base pairs, from the CGI start or end are used. Horizontal lines indicate the CGI, shore and shelf boundaries. The four panels show resorts with both types of ultrastable CpGs, only ultrastable unmethylated CpGs, only ultrastable methylated CpGs and no ultrastable CpGs. **(** **D** **)** Example resorts associated with the genes *MARS* (top; CGI chr12:57881750 to 57882035; ravine) and *TBX5* (bottom; CGI chr12: 114845861 to 114847650; not ravine) are depicted with individual sample methylation patterns as smoothed lines showing the methylation pattern of an individual across the resort. Resort feature positions are indicated by colored labelled bars. Lines indicate positions of 450 K probes assaying the resorts, ultrastable CpGs are highlighted with taller red lines. The histogram shows CpG density for bins of 50 bp on a scale of 0 to 0.2 CpG/bp. The gene track is extracted from UCSC Genome browser hg19 (refseq track).

Our initial analysis was to identify CpGs that have a consistent methylation state, across all available tissue, developmental stage and disease variation. To do this, we took advantage of the large amount of data available from the Illumina Infinium HumanMethylation450 BeadChip (450 K) [11]. The 450 K assays 485,577 CpGs in the human genome and is widely used in methylation studies, many of which are publicly available through the Gene Expression Omnibus (GEO; [23]). Careful quality control (see Methods) yielded a set of 1,737 samples from 30 different GEO series (a series typically reflects a single publication, [see Additional file 1: Table S1]), covering 26 tissue types and a wide range of conditions (Figure 1A and [see Additional file 1: Table S2]). We used a simple but stringent computational approach to identify candidate CpGs that were consistently methylated or unmethylated in all samples (see Methods). Based on this analysis, 974 CpGs were considered consistently methylated in every sample and 14,250 consistently unmethylated (Figure 1B, [see Additional file 1: Table S3 and Additional file 2]). Together, we refer to these as ‘ultrastable’ CpGs. These represent 3.1% of the CpG sites measured on the 450 K. A less stringent definition of ‘ultrastable’ would expand this set, but for our initial analysis we considered these as our starting pool.

One concern is that the apparent stability of a CpG might be a function of the platform and methodology. We therefore checked the methylation state of the ultrastable CpGs in the ENCODE reduced representation bisulfite sequencing (RRBS) data as validation. The 1.2 million CpGs measured in the ENCODE RRBS data include 17% of the sites assayed by the 450 K, including 5,063 (33%) of the ultrastable CpGs. Of the 121 ultrastable methylated and 4,942 ultrastable unmethylated CpGs of interest for which there was data available in ENCODE RRBS data, 80% and 98% were methylated and unmethylated, respectively, in 90% of RRBS samples [see Additional file 1: Figure S2]). The agreement of ENCODE RRBS data with our results was correlated with sequencing depth, so that higher-quality ENCODE sites tended to agree more closely with our methylation calls (that is, failures to verify tended to be poorly-covered sites in the ENCODE data). This suggests that the large majority of the ultrastable CpGs are not merely artifacts of the 450 K. We further tested whether these CpGs might be giving erroneous measurements due to unusual resistance or sensitivity to bisulfite conversion [24], which is used both by the 450 K and RRBS methods. We examined the status of CpGs assayed on the 450 k in methylation-sensitive restriction Enzyme Sequencing (MRE-Seq; which extracts unmethylated regions of the genome) and methylated DNA immunoprecipitation sequencing (MeDIP-Seq; which extracts methylated regions of the genome) data as neither technique involves a bisulfite conversion. We found that the ultrastable unmethylated CpGs have a significantly higher average read count in the MRE-Seq data than the other 450 K CpGs (*P* <0.001), confirming their stably unmethylated status. Similarly, the ultrastable methylated CpGs had a significantly higher average read count in the MeDIP-Seq than the other 450 K CpGs (*P* <0.01), confirming their stably methylated status. This analysis confirms that ultrastable CpGs are seen in both bisulfite-treated and non-bisulfite treated data [see Additional file 1: Figure S3]. Additionally, we examined the ultrastable CpGs in data sets that purposefully manipulated methylation, either by direct enzymatic treatment of the DNA, or by genetic knockout of DNA methyltransferases. This analysis showed that under appropriate conditions, the ultrastable sites can be measured in their opposite state. This suggests that there is no inherent problem with the ultrastable CpGs being measured at either methylation state, but that under a wide range of biological conditions, the CpGs are always in one state.

### Distribution of ultrastable cytosine guanine dinucleotide sites in the human genome

Because the ultrastable sites are consistent across a wide range of tissues, developmental stages and conditions, we hypothesized they would be of biological significance. Both classes of ultrastable sites tend to be near transcription start sites (TSS; *P* <0.001, *t*-test; accounting for the distribution of sites on the 450 K; [see Additional file 1: Figure S5]). Concomitantly, ultrastable CpGs tend to be associated with CGIs. Of all 450 K CpGs assayed, 62% are CGI-associated (in CGI, shore or shelf), while 95.5% of the ultrastable CpGs are CGI-associated. We also observed that ultrastable CpGs tend to be found in CGIs in groups of two or more, rather than in isolation, more often than expected by chance [see Additional file 1: Figure S6]). The ultrastable unmethylated CpGs are overrepresented in CGIs, rather than in shores and shelves. In contrast, ultrastable methylated CpGs are underrepresented in CGIs but overrepresented in CGI shelves [see Additional file 1: Figure S7]. This distribution is expected as CpGs in CGIs are generally unmethylated and those in the rest of the genome tend to be methylated. However, the extreme stability of these sites led us to hypothesize that the ultrastable CpGs might reflect other features of the CGIs they associate with, leading us to focus further investigation on CGIs. We leave a deeper analysis of the 1,134 non-CGI-associated ultrastable sites as a topic for future study.

### Profiles of regions containing ultrastable CpG sites

We stratified CGIs and their associated flanking shores and shelves into four categories based on the presence or absence of an ultrastable CpG. For brevity, following the terminology of [25], we use the term ‘resort’ to refer to the complex of a CGI and its flanking shores and shelves. We created a methylation profile for each resort category by aligning the CGIs, shores and shelves and plotting the mean methylation level of each CpG assayed in the resorts (see legend to Figure 1C and Methods). As shown in Figure 1C, an interesting pattern emerges. Resorts that contain at least one methylated and unmethylated ultrastable CpG (top panel) have a strikingly high contrast between the low methylation level of the CGI compared to the highly methylated shores and shelves. In comparison, resorts that lack ultrastable CpGs do not show this pattern (bottom panel), and such resorts the CGI can be either methylated or unmethylated, as can be the shores and shelves. Resorts that have only methylated or unmethylated ultrastable sites show an intermediate pattern (middle panels). To get a better sense of the correlation structure of methylation levels across single resorts, we visualized the data at sample-level for two characteristic resorts (Figure 1D). Generally, and in the examples shown, resorts with high contrast between CGI and shore/shelf show a very consistent pattern across samples whereas others do not. By analogy to the previously reported methylation ‘valleys’ and ‘canyons’ [14, 15], we refer to the sharp pattern shown in Figure 1D top panel as a ‘ravine.’ We note that ravines genomic positions do not overlap with canyons or valleys (in addition to being smaller; ravines average 785 bp of unmethylated region, canyons >3.5 kb and valleys >5 kb). Because gene body methylation has been previously reported to be positively correlated with gene expression [26, 27], we further tested whether the super-additive effect we observe could be explained by a ravine being equivalent to a CGI next to a highly methylated gene body. This appears to not be the case as ravines are symmetrical with respect to transcription direction, and ravines can be found away from gene bodies [see Additional file 1: Figure S8]. A further extensive comparison of ravines to a number of previously-defined methylation domain types shows that ravines represent a novel aspect of the methylome [see Additional file 1: Table S4]. To confirm our findings were not due to some idiosyncrasy of the set of 450 K samples or the parameters we used to define ravines, we tested whether the ravines had the same properties on an additional set of 757 samples of similar variety, which became available after we started our study [see Additional file 1: Table S5 and Figure S9]. The results show that the CGIs we classify as ravines, whether uniformly unmethylated or ‘other,’ have the same features in the new data set, strongly supporting the idea that ravines are stable features of these genomic regions.

### Ravines are associated with active transcription

**Figure 2:**
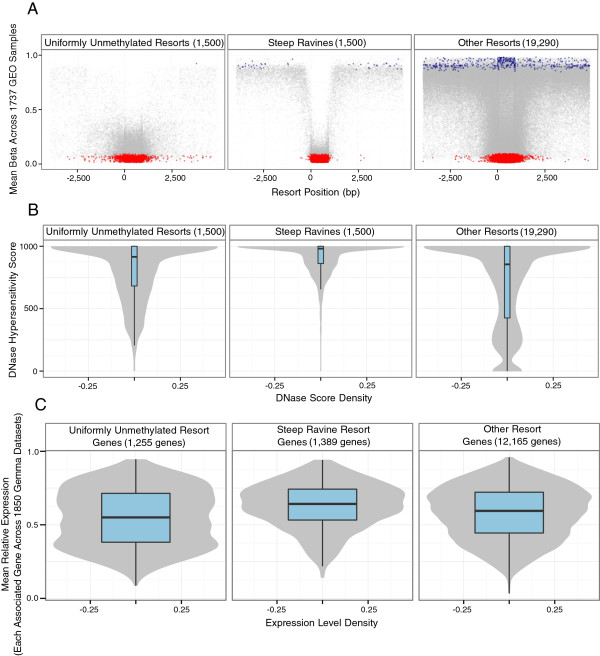
**Ravines are associated with higher transcriptional activity. (A)** Resorts are classified based on steepness, with the steepest 1500 resorts forming the steep ravine class and the least steep unmethylated resorts forming the uniformly unmethylated class. **(B)** Distribution of DNase sensitivity scores for each resort class. **(C)** Distribution of gene expression levels of all genes associated (5’, promoter or intragenic) with a CGI in the uniformly unmethylated, ravine or other resort classes. Density of DNase scores and expression levels are shown by the violin plots behind the box plots.

To identify ravines more comprehensively, we quantified the difference between the CGI and shore/shelf methylation levels (‘steepness’) for all 450 K resorts. In this manner we ranked all 27176 resorts assayed on the 450 K for their ‘ravine-ness’, independent of whether they contained an ultrastable CpG. As depicted in Figure 2A, the 1,500 resorts with steepest ravines (mean steepness 0.638) represent the most extreme ravine pattern (hereafter referred to as ‘steep ravines’) whereas the 1,500 unmethylated resorts with the lowest ravine steepness (CGI mean methylation <0.3 and mean steepness 0.097) show a more uniform pattern (hereafter referred to as ‘uniformly unmethylated resorts,’ mean methylation and CpG density of resorts [see Additional file 1: Table S6]).

To test whether the high methylation in the shores had an impact on the associated gene expression, we used the ENCODE DNase-sequencing data [2] as an indirect measure of non tissue-specific transcriptional activity. Unmethylated CGIs are generally associated with high transcriptional activity at their associated gene [7]. As expected, uniformly unmethylated resorts show significantly (*P* <0.001, Wilcoxon rank sum (Wilcoxon RS) test) higher DNase sensitivity than all other resorts. Interestingly, the steep ravines show significantly (*P* <0.001, Wilcoxon RS test) higher DNase sensitivity than the uniform resorts (Figure 2B). Since the main difference between the steep ravines and the uniformly unmethylated resorts is the highly methylated shores and shelves, it suggests that this high methylation on the edges of CGIs facilitates a transcriptionally permissive state. The relationship between high ravine steepness and high transcriptional activity is supported by analysis of a diverse set of microarray expression experiments (see Methods). Averaged across expression data sets, the expression of genes associated with steep ravines is significantly higher (*P* <0.001, *t*-test) than for the uniformly unmethylated resorts (Figure 2C).

We next tested whether the steepness of ravines was predictive of gene expression, beyond that which is possible using methylation level of the CGI alone, using a regression approach (see Methods). Gene expression variance (R^2^) explained by CGI methylation level alone is 4.6%, comparable to previous reports [28, 29] even though our expression and methylation data comes from different sources. Variance in expression levels explained by resort steepness alone is 3.4%. In combination, ravine steepness and CGI methylation level explain 9.8% of the expression variance, significantly greater than would be expected if they were purely additive (significant interaction, *P* <0.001, ANOVA).

The association of ravines with high transcriptional activity was also supported by ENCODE RNA polymerase II binding data (POLR2A; [2], [see Additional file 1: Figure S10]). Active transcription of ravine associated genes is not explained by changes in histone marks as ravine CGIs show no significant differences in the 12 histone marks measured by ENCODE ([2]; [see Additional file 1: Figure S11]). However, uniformly unmethylated resorts do show significant differences in H3k27me3 and H3k4me1 marks (*P* <0.001, Wilcoxon RS test; [see Additional file 1: Figure S11]).

### Ravines are associated with housekeeping genes

**Figure 3:**
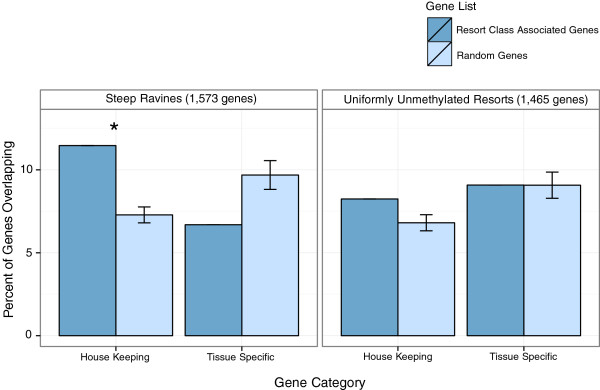
**Steep ravine genes are overrepresented for housekeeping genes.** Dark bars show the percent overlap of genes associated with steep ravines or uniformly unmethylated resorts with a list of housekeeping genes (2,064 genes) or tissue specific genes (2,293 genes). Light bars show mean overlap of housekeeping and tissue specific lists with random gene lists from all 450 K resort associated genes.

Taken together, the consistency of the ravine pattern, high DNase sensitivity and high associated gene expression, both across a variety of tissues and conditions suggests the genes associated with steep ravines are universally active in human cells. Indeed, we find that steep ravine-associated genes are significantly associated with a curated set of housekeeping genes (*P* <0.001, Fishers exact test; Figure 3), but not with tissue-specific genes [30]. In contrast the uniformly unmethylated resorts are not significantly associated with either set of genes (Figure 3). However uniformly unmethylated resorts are over represented for gene ontology (GO) groups for development and disease ontology (DO) groups for development associated diseases ([31]; [see Additional file 1: Tables S7 and S8]). Ravine associated genes had no significant enrichment for GO groups or diseases. Suggesting that ravines, which are maintained across tissues and conditions, may be regulatory features associated with the expression of ubiquitous genes, while uniformly unmethylated resort associated genes function in development.

## Discussion

Our contributions in this paper are twofold. First, we identified a subset of CpGs in the human genome that appears to be highly stable in their methylated or unmethylated state, across diverse developmental states and cell types. Second, we identified a subclass of CGIs that have an unusually high contrast between the methylation state of the CGI and the flanking shores. We found that such CGIs tend to be found near highly expressed genes. While the 450 K array only measures a subset of CpGs in the human genome, our results are consistent with a role for shore methylation in the regulation of genes which are ‘always on’.

The existence of CpGs with ultrastable methylation states reveals a previously undocumented feature of the human methylome. While some level of stability has been previously noted in differentiated somatic cells, the dramatic changes in methylation during development and differences between tissues [32] suggested that much of the methylome is dynamic. In contrast, our analysis suggests that a subset of the human methylome is highly stable across differentiated cell types, cancer cells, embryonic stem cells, induced pluripotent stem cells, trophoblasts and germ cells. The consistency of the CpGs across our developmental and germ cell samples suggests the state of the sites we found to be ultrastable are established early in development and then maintained in all studied differentiated tissues.

We cannot rule out the possibility that some of the ultrastable CpGs we identified will have a different state in some cell type or physiological state not yet examined. However, the data set we have assembled covers many of the states previously identified with variability, including between tissues [22], developmental states [9] and diseases [13, 33]. Indeed, we suspect there are many other CpGs in the human genome that show unusual stability but not revealed by our study. Our analysis used a very stringent threshold, disallowing even single exceptions; additional CpGs are ‘nearly stable.’ Furthermore the 450 K array does not assay most of the CpGs in the genome, some of which are likely to also be ultrastable. As there are only a few samples of certain tissue types, we could not assess the potential existence of tissue-specific ultrastable CpGs. Future experiments to assess additional CpGs and larger numbers and varieties of samples will help further elucidate the scope of ultrastable CpGs.

Our association of ultrastable CpGs with TSSs and resorts (94.5% in resorts) agrees with the previous observations that differentially methylated (that is, dynamic) regions are primarily located far from the TSSs, outside of resorts [22]. However, we note that the 450 K array is biased towards resort CpGs. The small subset of ultrastable CpGs we observe that are not in resorts (5.5%) hints that many other ultrastable CpGs may be outside resorts. Because most of the CpGs we identified are in or near resorts, we focused our analysis on their potential roles in resort function. Regarding the ravine pattern, we note that some degree of contrast between shores and CGIs is expected: the majority of CpGs are known to be methylated, with CGIs being the exception. However, we show that this contrast is not observed in all resorts, and is particularly striking in resorts that also contain ultrastable unmethylated CpGs. The association of steep ravines with higher gene expression levels, high DNase I sensitivity and high POLR2A occupancy provides a novel and biologically meaningful classification of human CGIs that complements earlier efforts [5].

In our study (as in many others), we attempted to relate the methylation state of a region to the expression level of nearby genes. However, it is not clear how to tell if a CGI is in a position to influence (or be influenced by) a gene. The examination of ravines may provide some insight. The classic association of genes with CGIs is based on the presence of a CGI in a gene’s 5′ promoter. This association is sufficiently strong that it was originally used to annotate human genes [34]. However, many CGIs do not appear to function as 5′ gene promoters ([35]; [see Additional file 1: Figure S12]). In contrast, the ravine CGIs are more strongly overrepresented in 5′ promoter regions of genes. Thus ravines fit the classic CGI archetype: an unmethylated CGI in 5′ promoter of a highly expressed gene. Ravines share an additional feature in common with the classical CGI, an association with housekeeping genes [36, 37]. The image of unmethylated 5′ promoter CGIs leading to gene expression may be more specific to ravines and not true for resorts and CGIs in general. A stable ravine pattern at many 5′ promoters supports the emerging idea that it is crucial to examine non-promoter CpGs and CGIs in differential methylation analysis as non-promoter regions may have more dynamic methylation than 5′ promoter regions.

While CGIs are the classic unit of focus for human methylation studies, other groups have focused on identifying other types of methylation domains [14–18] that have some overlap with CGI classes we identified. Specifically, uniformly unmethylated resorts (non-ravines) are encompassed by canyons and valleys [14, 15] more than other resorts, suggesting that uniformly unmethylated resorts, canyons and valleys may be related domains [see Additional file 1: Table S4]. Subsets of canyons and valleys lack H3k27me3 similar to uniformly unmethylated resorts. Additionally, uniformly methylated resorts, canyons and valleys are all enriched for genes which function in development. Thus uniformly unmethylated resorts are confirmation of canyons and valleys as features of the methylome in a greater variety of tissues. Ravines on the other hand minimally overlap with canyons, valleys or most other previously defined methylation domains. Additionally ravines show no obvious relation to histone marks. Other regulatory mechanisms are likely be involved which explain ravine association with stable and high gene expression.

One of our observations is that across a wide range of tissue types and developmental stages, DNA methylation flanking CGIs is positively correlated with gene expression, especially when the CGI has a very low methylation level. Previously, positive correlations between shore methylation and gene expression have been reported in some studies. Hansen *et al*. [38] and VanderKraats *et al*. [39] found tissue-specific ravine-like patterns emerging between cancer and healthy states as a differential methylation signature. This suggests that ravines might not just be a static feature associated with housekeeping genes, but one that can be generated under different conditions. While our manuscript was under review, Lou *et al*. [40] reported gene body methylation changes associated with increases in gene expression, a pattern that may also have a relationship to the ravines we observed. Although the association they saw was specifically in blood and limited to a family trio, it is further evidence that ravine-like patterns are positively correlated with gene expression. Another potentially relevant study showing a pattern similar to our ravines, from Wu *et al*. [41], found that the shores and shelves of unmethylated 5′ promoter CGIs, are associated with high Dnmt3a activity in the mouse genome. Wu *et al*. also found Dnmt3a- shore and shelf DNA methylation is associated with increased gene expression. We hypothesize that the regions identified by Wu *et al*. may correspond to ravines, but we were unable to confirm this with the information available. We speculate that possible Dmnt3a activity at steep ravines’ shores and shelves could function to antagonize the binding of transcriptional repressors. The previous work on Dnmt3a binding at gene promoters also found that shore and shelf methylation in proximal promoters antagonized polycomb protein-binding [41]. Interestingly, uniformly unmethylated resorts had a higher association with polycomb binding sites than steep ravines ([42]; [see Additional file 1: Polycomb Binding Sites]). On the other hand, we did not find evidence that ravines are associated with low H3k27me3, as would be predicted from polycomb binding inhibition [see Additional file 1: ENCODE Histone Modifications]. To resolve the function of ravines, it will be important to further explore their relationship with polycomb binding and other regulatory mechanisms.

An alternate model for ravine function is that transcription factors that bind methylated CpGs could be directly affected by shore and shelf methylation. However, most studies of methyl-CpG-binding proteins show they function to repress gene expression, agreeing with the classical model of any methylation in promoters being repressive [7]. There is, however, recent evidence of the methyl-CpG-binding protein MeCP2 having transcription activating function at promoters with methylated CpGs [43]. A model where MeCP2 binds methylated shores at gene promoters and performs its transcription activation function could explain the association of methylated shores and shelves with high gene expression.

## Conclusions

In summary, ravines are a novel subset of CGIs, distinct from previously identified methylome domains. The ultrastable CpGs and ravine consistency across samples suggests they are stable component of the human methylome. While the ravines suggest that CGI shore methylation is stably associated with high gene expression, other work has shown some CGI shores methylation to be highly dynamic. Both results support the overall importance of shores for gene expression. The presence of ravines in the 5′ gene promoters of many actively transcribed genes supports a complex role for methylation in both activating and repressing expression.

## Methods

### Data collection

As of 30 April 2013, 58 unique sample series run on the Illumina 450 K platform (GPL13534 or GPL16304) were available in the Gene Expression Omnibus (GEO) [23]. Using the R Bioconductor package ‘GEOquery’ 2.26.2 [44], the series were collected and considered for quality control [see Additional file 1: Table S1]).

### Quality control

To qualify for inclusion in our study, samples had to have beta values for all 485,577 probes, disqualifying 19 series. An additional four studies that involved direct global manipulation (genetic or chemical) of DNA methylation were also removed (DNMT1; DNMT3b double knockouts or methyltransferase treatment). Five more series were considered unsuitable for the meta-analysis, for individual reasons, and removed (that is, mislabeled data, high amount of missing data in all samples, multiple arrays grouped together, *etcetera*; see Additional file 1: Individual Study Quality Control for details of data exclusion justifications). Within each study, individual samples were further assessed for quality. Eight samples with unusually high numbers of missing values (5 SD from the mean, corresponding to >0.4% or 1957 were removed.

### Ultrastable cytosine guanine dinucleotide calling

A three-component mixture model was fit to each series beta distribution using the R ‘mixtools’ package [45]. The mean was calculated for each component; μ +2sd and μ - 2sd were used as the unmethylated and methylated beta value thresholds, respectively, for each series separately [see Additional file 1: Figure S1]. For each sample, unmethylated and methylated probes were called based on the thresholds computed for the series. Typical thresholds were near beta values of 0.2 and 0.8. Probes that were scored as methylated or unmethylated in all 1,737 samples were deemed ‘ultrastable.’

### ENCODE confirmation of ultrastable cytosine guanine dinucleotides

Data from 102 ENCODE RRBS samples was collected from UCSC (Release 3 of ENCODE/HudsonAlpha RRBS data; [2]). In many RRBS studies, reads with <10 fold coverage [46, 47] are discarded; therefore, a ten-fold coverage cutoff was used on the ENCODE RRBS data. CpGs were considered methylated in ENCODE RRBS data if their percent methylation was >80 and unmethylated if the CpG percent methylation was <20 [see Additional file 1: Figure S2].

### Methyltransferase confirmation of ultrastable cytosine guanine dinucleotides

Four methyltransferase 450 K studies (DNMT1; DNMT3b double knockouts, methyltransferase inhibitor or methyltransferase treated) with a total 68 samples were available on GEO [see Additional file 1: Table S1]. The studies were excluded from the ultrastable site calling, and the states of the ultrastable sites were then checked in the 68 samples.

### MRE-Seq and MeDIP-Seq confirmation of ultrastable cytosine guanine dinucleotides

From the NIH Roadmap Epigenomics Mapping Consortium data [48] 7 MRE-Seq and 7 MeDIP-Seq samples were used from seven tissue types (GSM669604, GSM669614, GSM543007, GSM543021, GSM669600, GSM669610, GSM543009, GSM543023, GSM707017, GSM941725, GSM428286, GSM456941, GSM543013, and GSM543027). Due to computational constraints, here we present data for chromosome 20 (analysis for other chromosomes is a work in progress). For the 10,379 450 K CpGs on chromosome 20 the reads covering a CpG seen in either technique were averaged across samples. The average number of reads across samples, from either technique, is used as the signal of methylated (MeDIP-Seq) and unmethylated (MRE-Seq) of a CpG. A Wilcoxon Rank Sum (Wilcoxon RS) test was used to test the significance of the difference between ultrastable sites on the array and non-ultrastable sites on the array.

### Ultrastable cytosine guanine dinucleotide characterization

To annotate the CpGs, we used three sources of information. The first was that provided by Illumina [11] and included UCSC CGI a CpG site is associated with and the CGI relation. CpG shores and shelves are defined by base pairs from the UCSC defined CGI start and stop coordinates. Shores are 2 kb from the CGI boundaries [10], and shelves are 2 to 4 kb from the CGI boundary [11]. The second annotation, available on GEO under GPL16304, contains additional probe annotations to those provided by Illumina under GPL13534 [49], including distance to nearest TSS. A Student’s *t*-test was performed to determine significantly different distance to TSS between all CpGs and ultrastable CpGs.

### Composite profile of resorts

The 27176 resorts have a range of lengths (minimum 201 bp, maximum 45,710 bp, mean 935 bp). To allow comparison of resorts, the position of a CpG in a CGI was converted to the CpG's relative position in a CGI of the mean CGI size (935 bp). As an example, a CpG 200 bp from the start of a 1,200 bp CGI would be shown at 155.83 bp from the start of the CGI in the composite plot. Conversion of CpG position to a relative value allowed comparisons of CGIs of varying sizes. Resort shores include all CpGs less than 2 kb from the CGI start or end. CGI shelves include all CpGs 2 to 4 kb from the CGI start or end. Since shores and shelves are fixed sizes CpG positions within shores and shelves are shown at their actual, not relative, distance from the CGI boundaries.

### Resort classifier based on ravine steepness

Steepness of a ravine was only calculated for those resorts which had at least one CpG measured on the 450 K array in each relevant part of the result (CGI, the north shore or shelf and the south shore or shelf; 22,290 resorts). Steepness was calculated as mean beta methylation level of the CGI CpGs subtracted from the mean beta methylation of the shore and shelf CpGs. Steep ravines were arbitrarily defined as those with the 1,500 highest steepness values. Uniformly unmethylated resorts were defined as those with a CGI mean methylation <0.3 and the 1,500 lowest steepness values.

### ENCODE DNase sensitivity data

ENCODE UCSC DNase clusters track (wgEncodeRegDnaseClusteredV2) from the University of Washington and Duke University were collected for 125 cells types [2]. DNase score for a CGI was calculated by taking the score for any DNase hot spot overlapping a CGI body. If multiple DNase hot spots overlapped a CGI, the scores were weighted by the amount of the CGI the DNase peak overlapped. Wilcoxon RS tests on the DNase data were performed among the three classes of resorts.

### Cytosine guanine dinucleotide island-to-gene associations

There are multiple methods of annotating a CGI with a gene association, including the annotation a CGI with closest gene TSS to the CpGs making up a CGI [49], the position of the CpGs making up a CGI in a gene’s body or promoter [11], or overlap of an entire CGI with a gene’s body or promoter [35]. Each yields a slightly different CGI to gene associations. Even with a given method, a CGI can end up associated with more than one gene [see Additional file 1: Figure S4]. For this study, an inclusive CGI to gene association was used. Genes that overlap a CGI in their promoter or gene body were considered associated with that CGI. An inclusive association was used because the exact role of CGIs and resorts in regulating gene expression is unclear. Using inclusive associations will hopefully capture any possible CGI effects on gene expression.

CGI were considered associated with a gene if the CGI is located in the gene body or in promoter region of a gene. Classifications of CGI in promoters and gene bodies were based of the [35] definitions. Refseq genes were downloaded from UCSC. For Refseq genes with multiple transcripts the longest form was used, to capture any possible intragenic functions. Non-coding RNA (ncRNA) annotations were collected from Ensembl [50]. The final list included 40,721 unique transcription units. There are 21,743 CGI on the 450 K array associated with 17,725 genes or ncRNA (39% intragenic CGI, 61% promoter CGI).

Although gene expression results are subject to noise from incorrect CGI to gene associations, DNase sensitivity data is independent of gene to CGI associations. DNase sensitivity data will capture the effects of methylation on transcriptional activity without absolute gene to CGI associations. Until CGI to gene associations are definite DNase sensitivity data will be valuable to pair with methylation for examining transcriptional activity.

### Gene expression data

Gene expression data from 2,021 GEO expression studies were assembled from the Gemma database [51], representing 97,388 samples and 34 tissue types. Expression information was available for 21,733 genes, 14,809 of which were associated with one of 22,290 450 K CGI (only those CGI in resorts previously classed by steepness were compared for expression). Student’s t tests on the gene expression data were performed among the three classes of resorts. Linear regression was done with 17,127 CGI (CGI with associated gene expression level and steepness class). Models were for expression variance with associated resort steepness and associated CGI mean methylation, and resort steepness and CGI methylation interaction. An F-test was used to show significant interaction of resort steepness and CGI methylation.

### Steep ravine-associated gene function

List of steep ravine- and uniform resort-associated genes are the same as those used with the gene expression data. One hundred random gene lists of the same length as the steep ravine and uniform resort gene association lists (1,573 and 1,465, respectively) were generated. Percent overlap of each random gene list and either the housekeeping or tissue-specific list was calculated. Mean overlap of the 100 random lists with the housekeeping and tissue-specific lists were taken as the expected overlap from comparison with the steep ravine and uniform resort gene lists. Fisher’s exact tests were performed between each random gene list overlap and steepness class gene lists.

We used the GO annotations of the 19,389 genes associated with the 450 K probes [11] and disease ontology (DO) terms from the Phenocarta database [31] for enrichment analysis. Enrichment of GO and DO groups in uniformly unmethylated resort- and ravine-associated genes using overrepresentation analysis was done in ErmineJ [52]. Statistical significance is reported as false discovery rates computed using the Benjamini-Hochberg method in ErmineJ. Also calculated are the multifunctionality scores of the ontology gene sets [53], as well as the *P* values corrected for multifunctionality.

## Electronic supplementary material

Additional file 1: **Supplementary Information.** Description of data: Additional analyses, figures and tables. (PDF 6 MB)

Additional file 2: **Ultrastable CpGs loci.** Description of data: Probe_ID is the 450 K CpG probe ID given by Illumina. State is the state of ultrastable CpG. Coordinate_37 is the Human Genome Build 37 position. Chromosome_37 is the Human Genome Build 37 chromosome. (TXT 737 KB)

## Abbreviations

CDMR: cancer-specific differentially methylated region
CGI: CpG island
CpG: cytosine guanine dinucleotide
cUMR: control unmethylated region
DO: disease ontology
FFPE: formalin-fixed paraffin-embedded
GEO: Gene Expression Omnibus
GO: gene ontology
LMR: low methylated region
LREA: long-range epigenetic activation
LRES: long-range epigenetic silencing
MeDIP-Seq: methylated DNA immunoprecipitation sequencing
MRE-Seq: methylation-sensitive restriction enzyme sequencing
ncRNA: non-coding RNA
POLR2A: RNA polymerase II
RDMR: reprogramming specific differentially methylated region
RRBS: representation bisulfite sequencing
TDMR: tissue specific differentially methylated region
TFBS: transcription factor binding site
TSS: transcription start sites
UMR: unmethylated region
Wilcoxon RS: Wilcoxon rank sum
450 K: Illumina Infinium HumanMethylation450 BeadChip.
