# Supplementary material for: Meta-analysis of human methylomes reveals stably methylated sequences surrounding CpG islands associated with high gene expression
Source: Epigenetics Chromatin. 2014 Oct 23;7:28. doi: 10.1186/1756-8935-7-28 (PMC4260796; doi:10.1186/1756-8935-7-28)
Supplement: Supplementary file 1 — Additional file 1: Supplementary Information. Description of data: Additional analyses, figures and tables. (PDF 6 MB) [file 13072_2014_333_MOESM1_ESM.pdf]

Supplementary Materials

Table of Contents

1. Individual Study Quality Control 2
2. Ultra-Stable CpG Confirmation 2
3. Chromosomal Distribution 2
4. Concurrence of Ultra-stable CpGs in CGIs 3
5. Ravines are not a by-product of gene body methylation 3
6. Ravine Confirmed in Independent Samples 3
7. ENCODE RNA Polymerase II Binding 3
8. ENCODE Histone Modifications 4
9. Ravine Independence from Previously Defined Methylome Domains 4
10. Resort Class CpG Density and Methylation Variability 5
11. CGI Categorization by Gene Relation 5
12. Polycomb Binding Sites 6
13. Supplemental References 7

1. Individual Study Quality Control

Some studies were flagged during initial quality control steps because of design features which makes them unsuitable for meta-analysis. GSE38271 (Lecher et al. 2013) contains 42 Formalin-Fixed Paraffin-Embedded (FFPE) samples. FFPE samples showed methylation totals beyond the standard deviation limit set for sample quality, while the untreated samples do not. Therefore GSE38271 was excluded from further analysis. GSE30338 (Turcan et al. 2012) was listed as having beta values provided. However, the data ranges from -4 and 4. Likely the values are M values, but out of caution we removed the series from the meta-analysis. GSE20945 (Tsai et al. 2012) was removed as the series is provided with multiple array data in one file. The structure would not easily fit into the collection script. GSE41273 (Alisch et al. 2013) was removed as it had a mean Pearson correlation of 0.023 with the other series, compared to an average of 0.96 between all other series. Though the cause of the low series correlation with GSE41273 is unclear, the series was removed from the meta-analysis.

2. Ultra-Stable CpG Confirmation

Of the final 1737 samples 750 had detection P values available to estimate the quality of ultra-stable probes. Of 485577 probes only 43388 had a detection p value >0.001 in 1% of samples. Of the 43388 lower quality probes only one is an ultra-stable probe. Therefore ultra-stable probes are not being called ultra-stable because they are generally poor performing.

An additional control was to examine the proportion of SNPs in ultra-stable 450K probes and SNPs at the CpG assayed by the 450k probe using the Price *et al.* annotation. The number of SNPs in ultra-stable probes was similar to the number in all probes, and fewer SNPs at ultra-stable CpG sites (Price et al. 2013) (Supplementary Figure S13).

3. Chromosomal Distribution

Ultra-stable CpGs are evenly distributed across autosomes, but not on the sex chromosomes. There are no ultra-stable CpGs on the Y chromosome and only 4 unmethylated CpGs on the X (Supplementary Figure S14).

4. Concurrence of Ultra-stable CpGs in CGIs

Ten sets of 15244 CpGs were randomly sampled from all 450K CpGs. The average number of randomly selected CpGs present within each CGI was taken as an expected distribution of 15224 CpGs across the CGIs to compare to the distribution of the 15224 ultra-stable CpGs. Fewer CGIs had only one ultra-stable CpGs than expected by chance and more CGIs had 2 or more ultra-stable CpGs than expected by chance (Supplementary Figure S6).

5. Ravines are not a by-product of gene body methylation

Using our CGI-to-Gene associations, we separated all resorts in 4 categories: “5 Prime” where one shore/shelf and the CGI core are located 5’ of the gene, but the other shore/shelf are overlapping the gene body, “Gene Body” where the whole resort is contained within the gene body, “3 Prime” where one shore/shelf are overlapping the gene body but the CGI core and the other shore/shelf are downstream the gene, and “Intergenic” where the whole resort is away from any gene. Ravines are symmetrical in 5 prime and 3 prime regions and do not show more methylation where they overlap with the gene body (Supplementary Figure S8). As well ravines exist entirely separate from gene bodies in Intergenic regions. So ravines are not simply a by-product of higher gene body methylation.

6. Ravine Confirmed in Independent Samples

The original 1737 sample were collected for all 450K studies available before April 30th 2013. Between April 30th 2013 and July 29th, 2013 additional samples were collected as an independent sample set for ravine confirmation. A total of 27 new series were available and 15 passed quality control leaving 757 samples to confirm the ravine pattern (Supplementary Table 5). The new samples were from a similar variety of tissues as the original samples (Supplementary Figure S9A). The previously defined uniformly methylated resorts and steep ravines show the same pattern in the new 757 samples, and confirm the existence of the ravine pattern at resorts (Supplementary Figure S9B).

7. ENCODE RNA Polymerase II Binding

ENCODE collected transcription factor binding site (TFBS) information for 161 transcription factors in 91 cell types (ENCODE Project Consortium 2012). We used the Chip-seq clusters V3 data from UCSC for our analysis. We scored each 450K CGI for all 161 factors (data not shown). While many of the transcription factors could be of interest in comparison between resort classes, we focused on the RNA polymerase subunit POLR2A as confirmation that ravines are associated with

higher transcriptional activity. In fact ravine CGIs did show significantly higher POLR2A scores ($p<0.001$, Wilcoxon RS test).

8. ENCODE Histone Modifications

ENCODE collected histone modification data for 12 marks in 46 cells types with similar variety of cell types to our 450K methylation data (ENCODE Project Consortium 2012). We scored each CGI for each histone mark by overlapping histone peaks with CGI. Different scores for a histone mark from different ENCODE samples in one CGI were weighted by percent overlap with the CGI and then averaged. Only two histone marks were significantly different between resort classes, H3K27me3 and H3K4me1. Some differences in histone mark scores between resort classes were significant but with a very small actual difference in mean (i.e H3K4me3 scores were significantly different between uniformly unmethylated and ravine CGIs ($p<0.001$, Wilcoxon RS test) but the actual difference in mean scores was only 500). Therefore only comparisons with a difference in mean >3000 were considered truly significant. Uniformly unmethylated resorts have significantly greater and significantly less H3K27me3 and H3K4me1 marks, respectively ($p<0.001$, Wilcoxon RS test; Supplementary Figure S11). H3K27me3 being a repressive mark and H3K4me1 being a mark of regulatory elements or TSS (ENCODE Project Consortium 2012) does not explain, and is somewhat opposite to the gene expression and DNase sensitivity results. The role of ravines in permitting gene expression may therefore involve other histone modifications or perhaps transcription factor binding.

9. Ravine Independence from Previously Defined Methylome Domains

We compared ravines to several previous classifications of sites. For comparison to human data, we used UCSC LiftOver (Hinrichs et al. 2006) to convert mouse genome coordinates or previous human genome builds to corresponding orthologous sites in hg19. The comparisons are summarized in Supplementary Table 4, with details in the following paragraphs.

Irizarry *et al.* assembled a list of tissue specific (TDMR) and cancer specific (CDMR) differentially methylated regions. Irizarry *et al.* defined shores by finding shores to be more frequently dynamic than CGIs. We assessed the overlap between TDMR and CDMR and ravines and uniformly unmethylated shores and observed that ravine shores are more stable and less frequently overlap with differentially methylated shores (Supplementary Table S4). In call cases regions or domains were considered overlapping if any bp were similar between them. Doi *et al.* assembled reprogramming specific (RDMRs) differentially methylated regions. Both ravines and uniformly unmethylated resorts

are underrepresented for RDMRs (Supplementary Table S4). Combined, these results show that, as expected given their apparent stability, ravines are not often differentially methylated.

Canyons defined in Jeong *et al.* and valleys defined in Xie *et al.* frequently encompass uniformly methylated resorts, but rarely steep ravines (Supplementary Table S4). Jeong *et al.* reported "control unmethylated regions" (cUMRs) which show a large overlap with all resorts on the 450k, which could be due to the resorts having generally unmethylated CGIs (Supplementary Table 4).

Stadler *et al.* identified low methylated and unmethylated regions (LMR, UMR) in the mouse genome. There is little overlap between LMRs and the orthologous human regions. UMRs are expected to overlap with CGI and our results confirm UMRs tend to be orthologous to unmethylated CGIs in human, but not specifically one class of resort as we have defined (Supplementary Table 4).

We also assessed the overlap of the resort classes with LREA and LRES domains (Bert et al. 2013; Coolen et al. 2010) and observed no overrepresentation in ravines (Supplementary Table S4).

10. Resort Class CpG Density and Methylation Variability

Shore methylation, or lack of, in uniformly unmethylated resorts is not due to a lack of CpGs or 450K probes in the uniformly unmethylated shores and shelves. In fact shores in uniformly unmethylated resorts are significantly more CpG-dense than ravine and all other resort shores ($p<0.001$, Wilcoxon RS test; Supplementary Table S5). Ravine CGIs are significantly more CpG dense than and uniformly unmethylated and all other CGIs ($p<0.001$, Wilcoxon RS test; Supplementary Table S5). While ravines are CpG dense, there is however little obvious relation of ravines to a previous CGI classification based on CpG density (Saxonov et al. 2006). Only two ravines are within 10kb of a high CpG content CGI as defined by Saxonov et al. 2006. Additionally uniformly unmethylated CGIs show greater variability (greater standard deviation) in methylation levels than other resort CGIs (Supplementary Table S5).

11. CGI Categorization by Gene Relation

While CGIs are classically thought of as gene promoter features, only ~45% of CGIs are located in the promoter region of a Refseq gene (Maunakea et al. 2010). We assigned each CGI to a class based on its proximity to Refseq or ncRNA genes using the Maunakea *et al.* definitions for promoter, intragenic, intergenic, and 3' CGIs. CGIs with multiple annotations (3368 CGIs, i.e. 3' to one gene and promoter to another) were excluded, leaving 25549 classified CGIs (Maunakea et al. 2010).

Sampling 100 ravine, uniformly unmethylated, and all other resorts 25 times, we were able to perform a Wilcoxon RS test on the relation of each resort type to genes. We found that steep ravine CGIs are significantly overrepresented in promoters and underrepresented in gene bodies (Supplementary Figure S12).

12. Polycomb Binding Sites

Lee *et al.* defined 3710 polycomb binding sites in human embryonic stem cells using ChIP-seq with the SUZ12 subunit of polycomb repressive complex 2 (Lee et al. 2006). The SUZ12 sites were significantly closer to uniformly unmethylated resort CGIs ($p<0.001$, Wilcoxon RS test). Steep ravine CGIs were not significantly different in distance from SUZ12 sites than all CGIs.

13. Supplemental References

51. Lechner M, Fenton T, West J, Wilson G, Feber A, Henderson S, Thirlwell C, Dibra HK, Jay A, Butcher L, Chakravarthy AR, Gratrix F, Patel N, Vaz F, O'Flynn P, Kalavrezos N, Teschendorff AE, Boshoff C, Beck S: **Identification and functional validation of HPV-mediated hypermethylation in head and neck squamous cell carcinoma.** *Genome Med* 2013, **5**(2):15.

52. Turcan S, Rohle D, Goenka A, Walsh LA, Fang F, Yilmaz E, Campos C, Fabius AW, Lu C, Ward PS, Thompson CB, Kaufman A, Guryanova O, Levine R, Heguy A, Viale A, Morris LG, Huse JT, Mellinghoff IK, Chan TA: **IDH1 mutation is sufficient to establish the glioma hypermethylator phenotype.** *Nature* 2012, **483**(7390):479-483.

53. Tsai HC, Li H, Van Neste L, Cai Y, Robert C, Rassool FV, Shin JJ, Harbom KM, Beaty R, Pappou E, Harris J, Yen RW, Ahuja N, Brock MV, Stearns V, Feller-Kopman D, Yarmus LB, Lin YC, Welm AL, Issa JP, Minn I, Matsui W, Jang YY, Sharkis SJ, Baylin SB, Zahnow CA: **Transient low doses of DNA-demethylating agents exert durable antitumor effects on hematological and epithelial tumor cells.** *Cancer Cell* 2012, **21**(3):430-446.

54. Alisch RS, Wang T, Chopra P, Visootsak J, Conneely KN, Warren ST: **Genome-wide analysis validates aberrant methylation in fragile X syndrome is specific to the FMR1 locus.** *BMC Med Genet* 2013, **14**:18-2350-14-18.

55. Hinrichs AS, Karolchik D, Baertsch R, Barber GP, Bejerano G, Clawson H, Diekhans M, Furey TS, Harte RA, Hsu F, Hillman-Jackson J, Kuhn RM, Pedersen JS, Pohl A, Raney BJ, Rosenbloom KR, Siepel A, Smith KE, Sugnet CW, Sultan-Qurraie A, Thomas DJ, Trumbower H, Weber RJ, Weirauch M, Zweig AS, Haussler D, Kent WJ: **The UCSC Genome Browser Database: update 2006.** *Nucleic Acids Res* 2006, **34**(Database issue):D590-8.

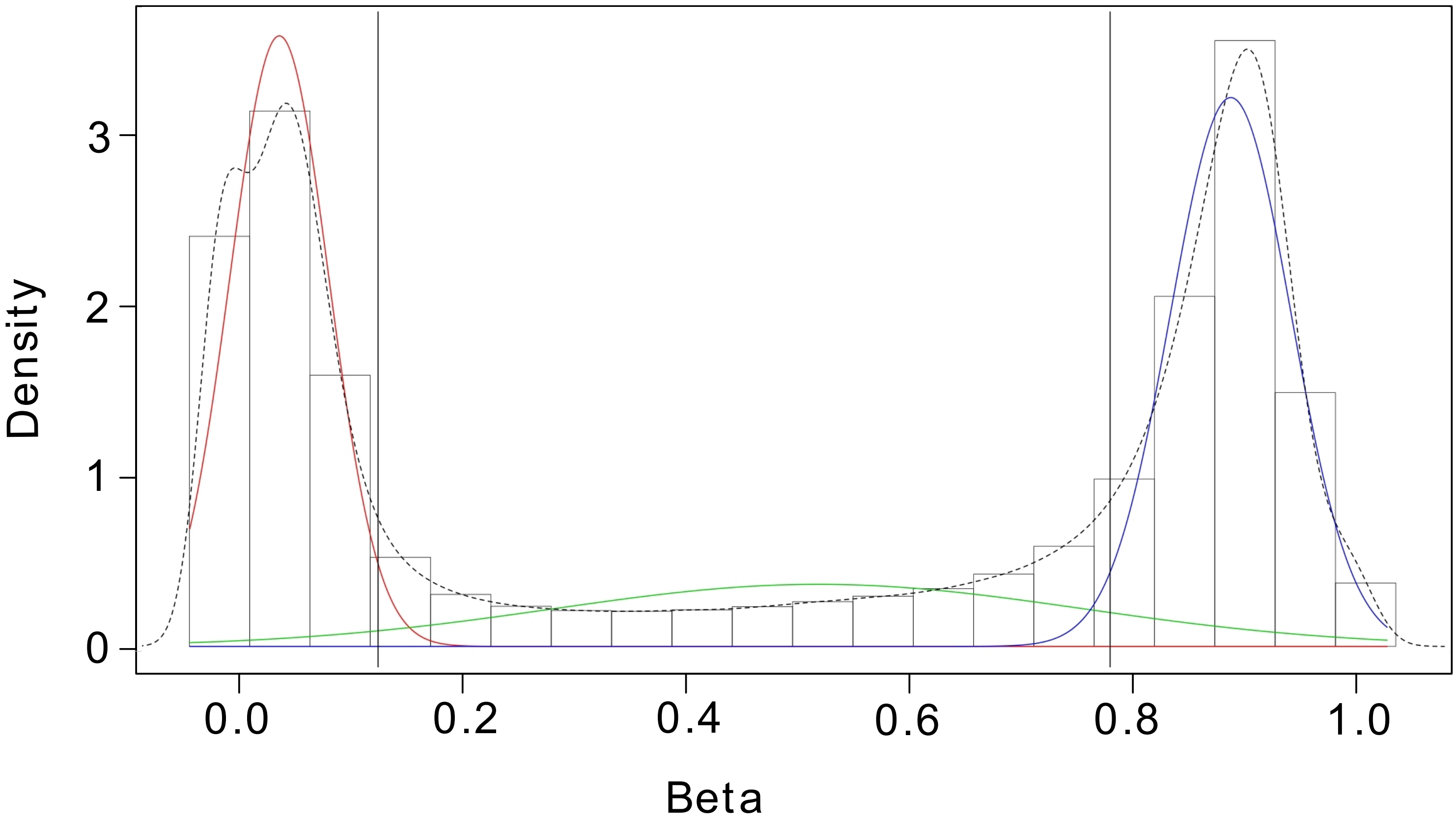

Supplementary Figure S1. Methylated and unmethylated thresholds were called separately for each series based on the series beta distribution. The histogram and broken line show the distribution of beta values for series GSE42118 as a representative example. Solid lines are the 3 fitted Gaussian components of the distribution (red unmethylated peak, blue methylated peak and green partially methylated peak). Vertical black lines indicate 2sd away from the unmethylated and methylated component means which were used as the methylation state thresholds.

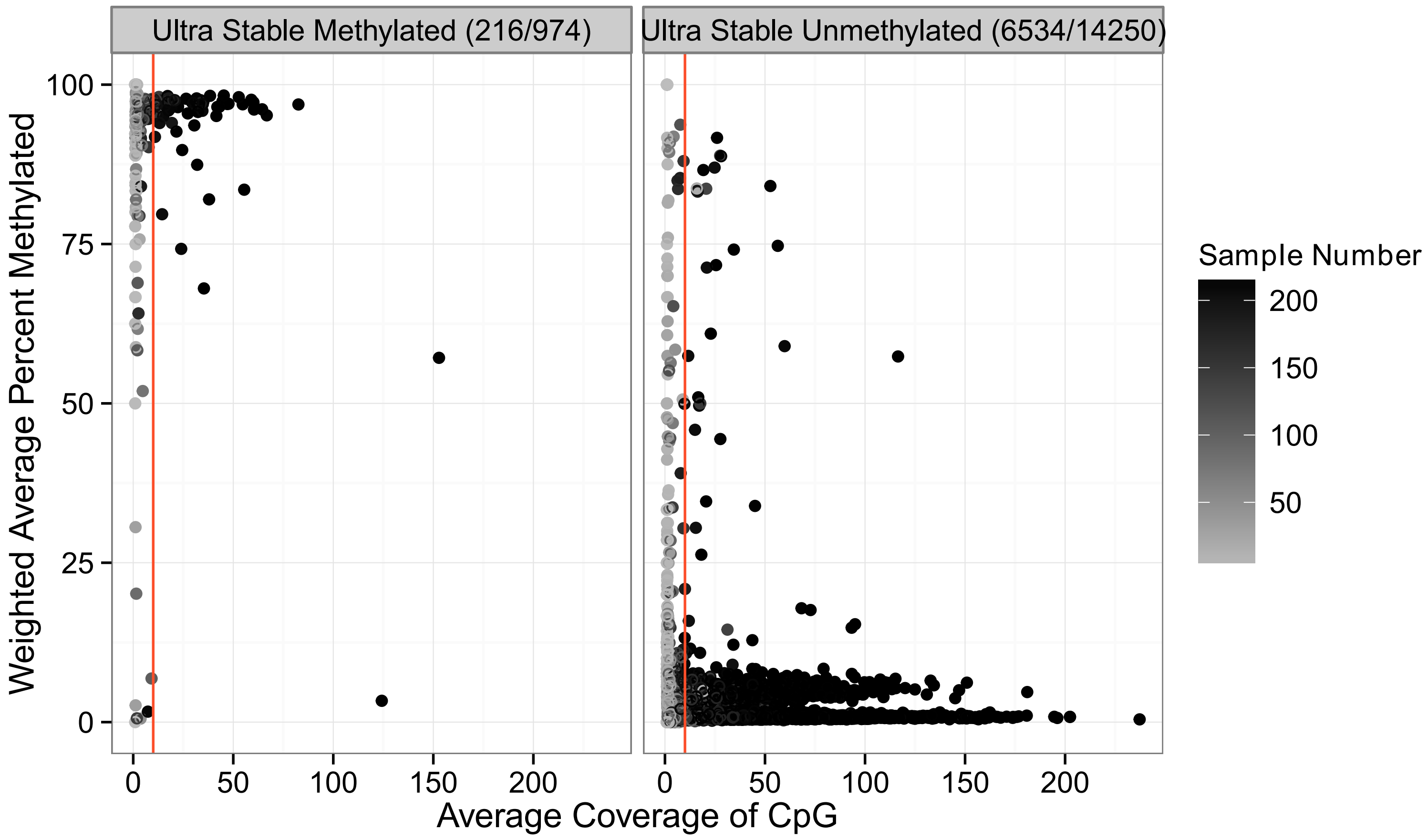

Supplementary Figure S2. Ultra-stable CpG states were confirmed by ENCODE RRBS data. Panels show the ultra-stable methylated CpGs (left panel; 216 captured in RRBS) or unmethylated CpGs (right panel; 6534 captured in RRBS) average methylation level across samples, weighted by the CpG coverage in a sample. Average coverage was cut o at 10 fold for state confirmation, indicated by vertical marker. Point color indicates how many runs of RRBS the CpG was measured (max 216, 102 samples with replicates), so the darker the point the more confidence.

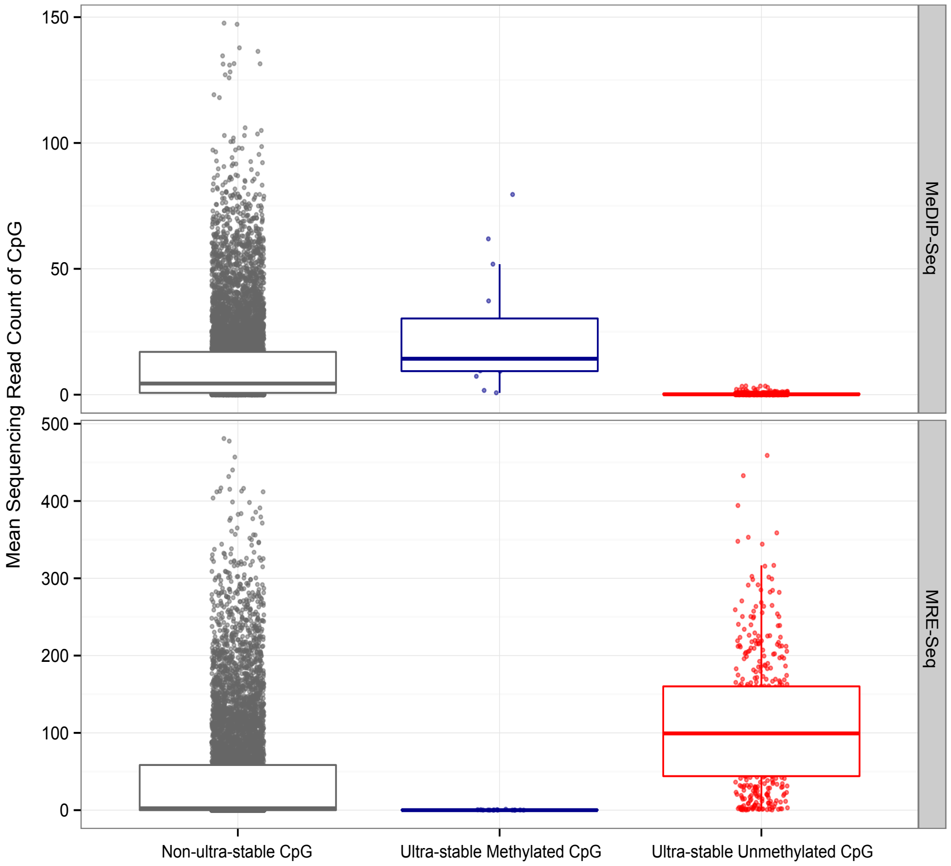

Supplementary Figure S3. Chromosome 20 Ultra-stable CpGs are confirmed in MREseq and MeDIP data. Boxplots show the distribution of read counts from (A) MREseq (7 samples, technique captures DNA fragments with unmethylated CpGs for sequencing) and (B) MeDIP (7 samples, technique captures DNA fragments with methylated CpGs for sequencing) averaged across samples. The non-ultra-stable CpGs are the CpGs measured by the 450K which were not seen as ultra-stable in the original 450K data analysis.

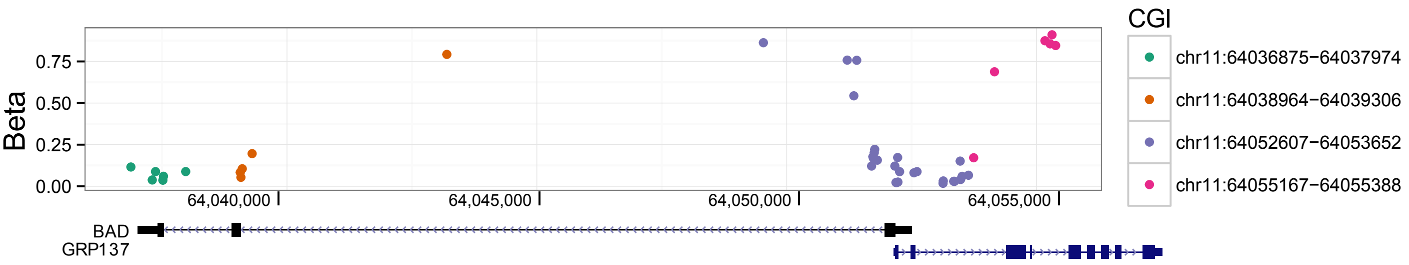

Supplementary Figure S4. CGI to gene annotations are not all one-to-one. A representative CGI (chr11:64052607-64053652) with multiple possible gene associations. The CGI is located in both genes promoter regions, by the definition used in this paper.

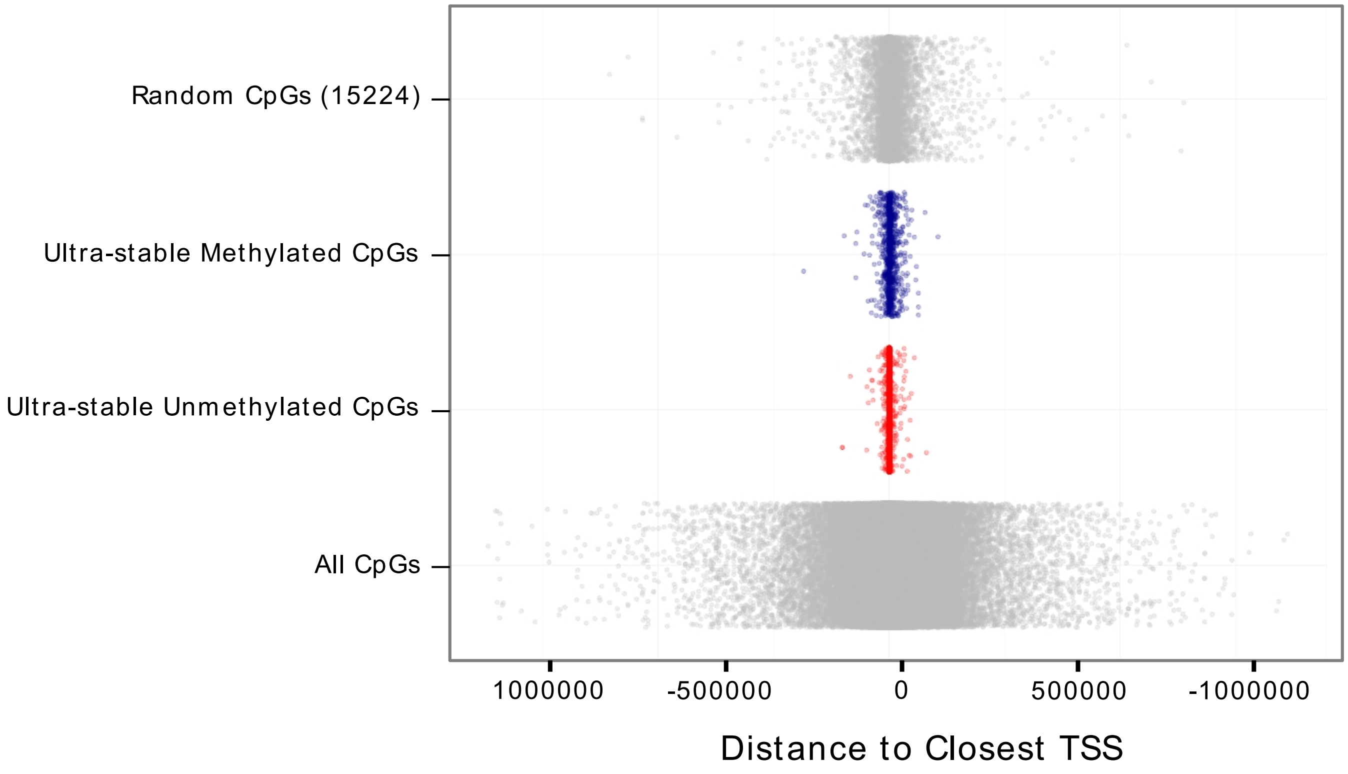

Supplementary Figure S5. Ultra-stable CpGs are located closer to TSSs. Distance of CpGs to the closest TSS (regardless of gene directionality) is shown.

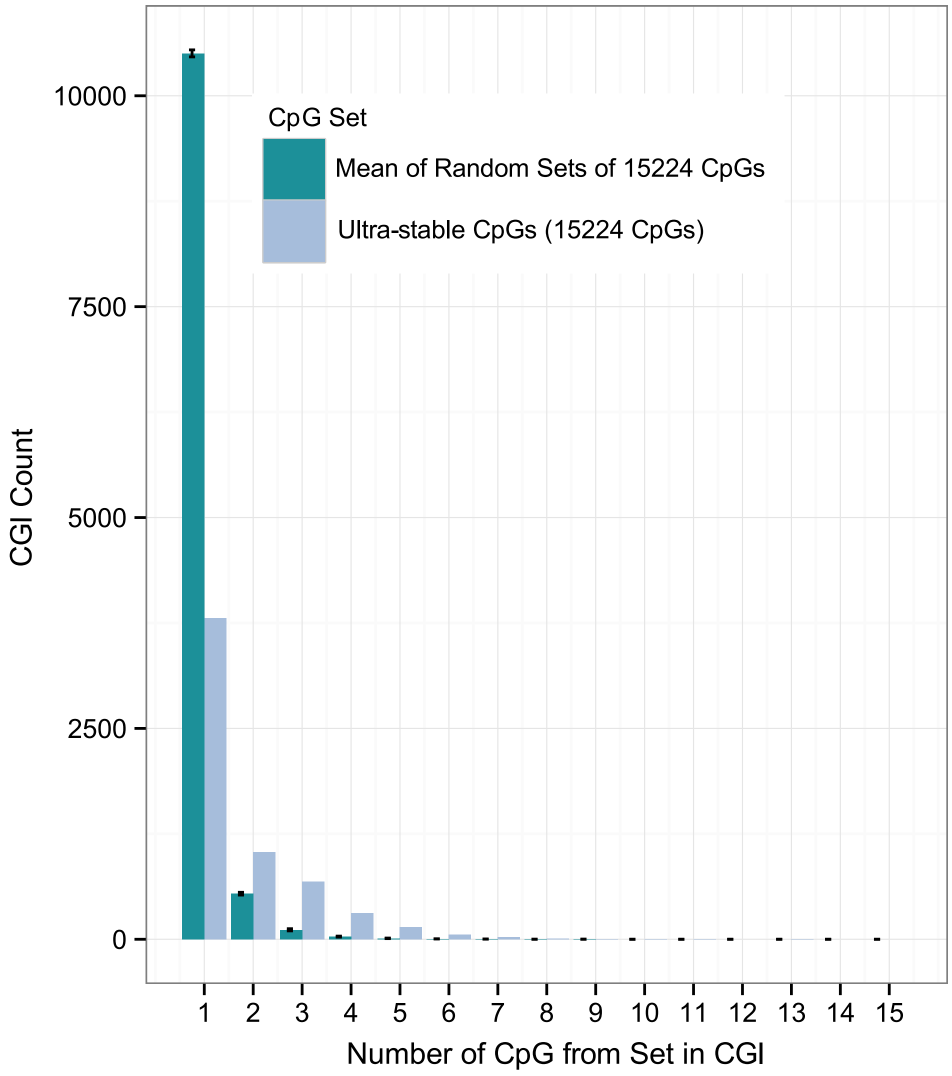

Supplementary Figure S6. Ultra-stable CpGs co-occur more than expected by chance. Bars show counts of CGI containing from 1 to 15 CpGs from the set of ultra-stable CpGs (grey) or random sets of CpGs (green; means of 10 sets are shown with standard error).

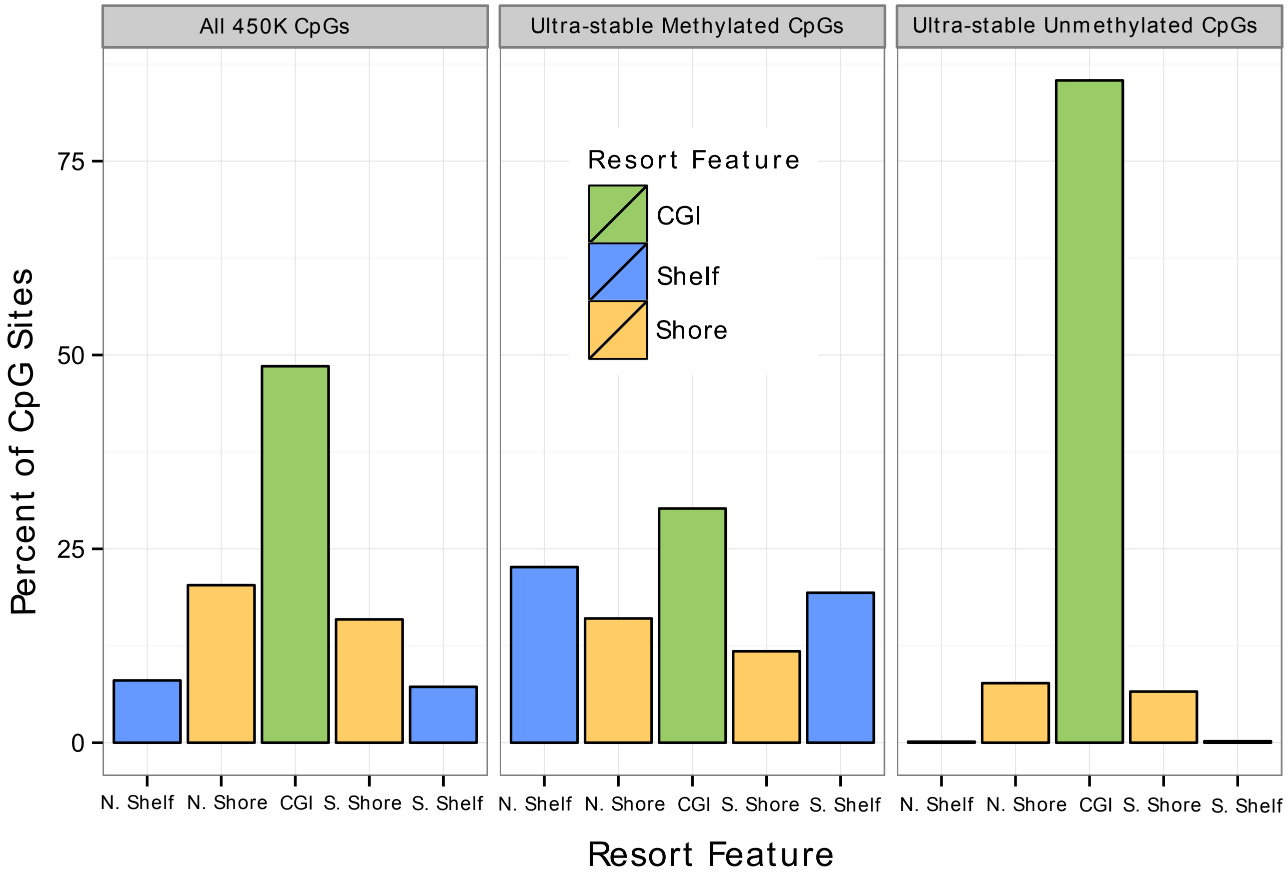

Supplementary Figure S7. Ultra-stable CpGs follow known trends of methylated and unmethylated CpGs. Proportions of CpGs in each resort feature from both CpG ultra-stable types as well as all CpGs on the 450K.

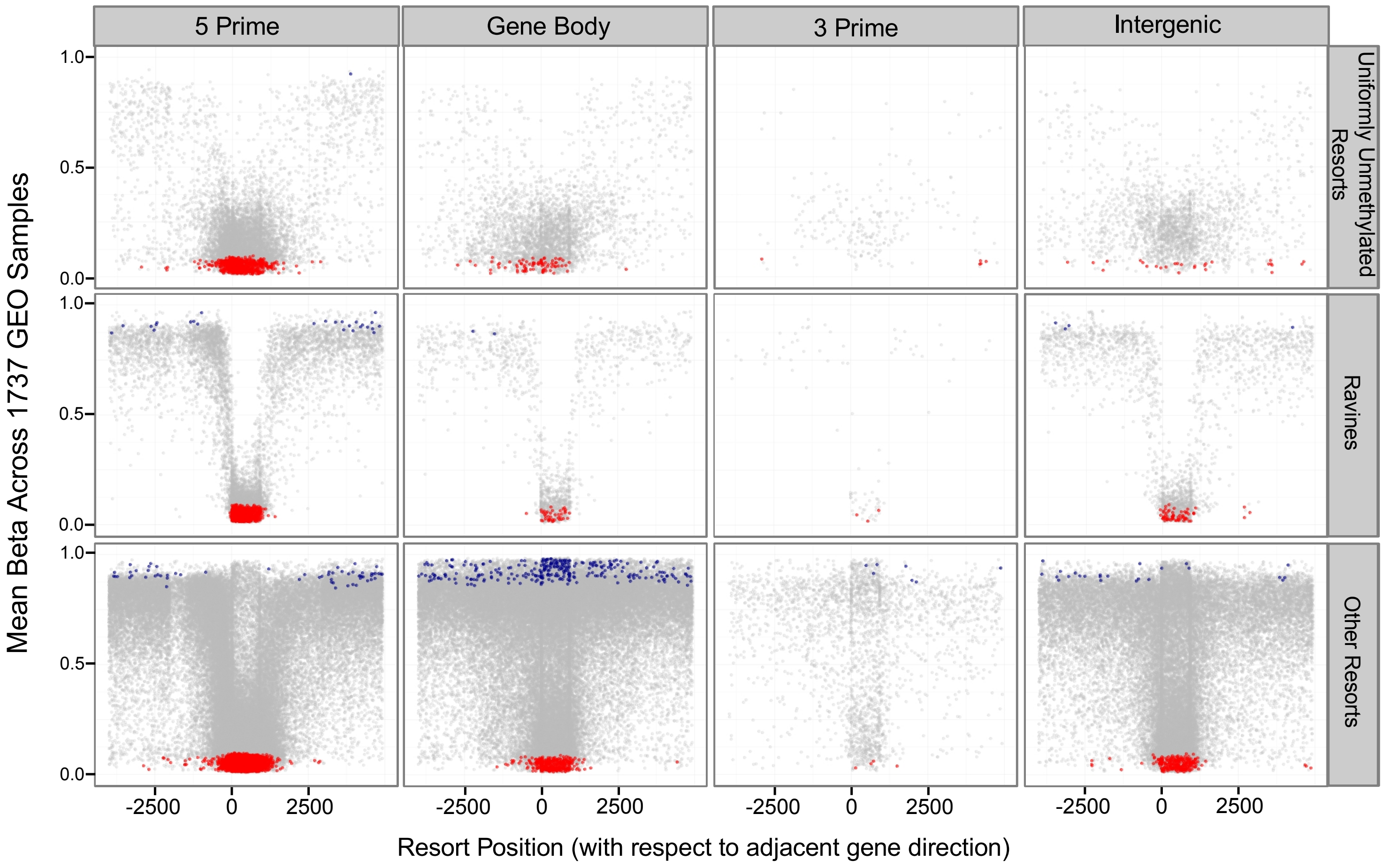

Supplementary Figure S8. The ravine pattern is not due to the shores/shelves being located within highly methylated gene bodies. Columns of plots separate the resorts by position of a resort in relation to the associate gene. Rows of plots separate resorts by class our defined classes. Resort position is distance from the CGI start.

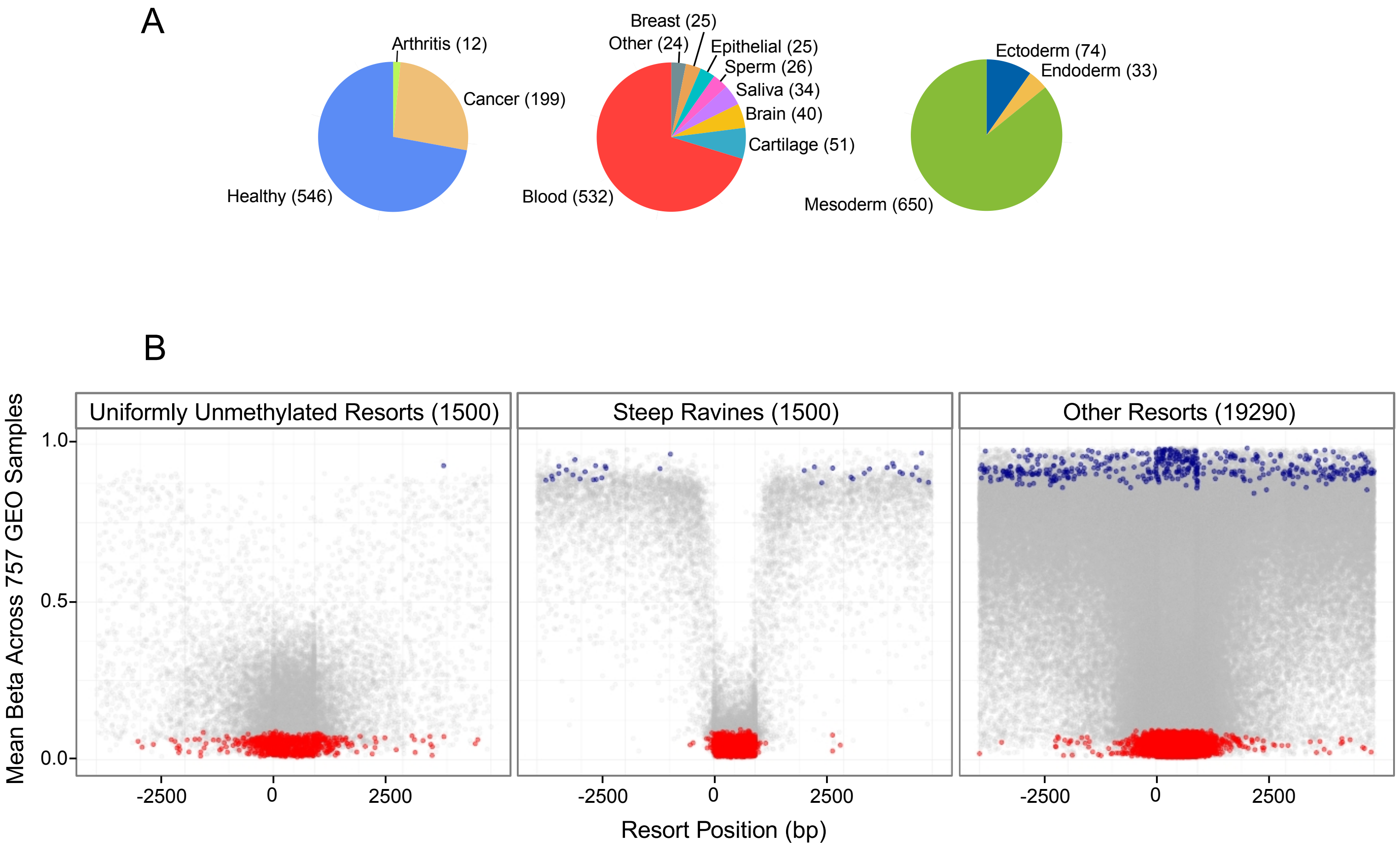

Supplementary Figure S9. Ravines are confirmed in independent 450K samples. (A) Counts of new 450K samples disease tissue and germ layer samples used in analysis. (B) Resorts are classified based on steepness in the original samples but mean methylation levels shown here are in the new 757 samples.

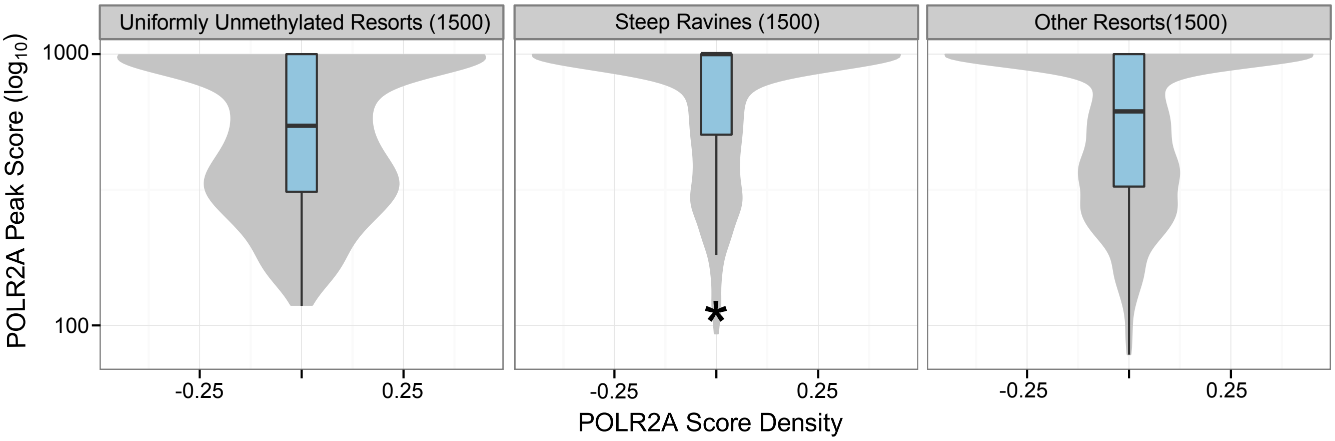

Supplementary Figure S10. RNA Polymerase II binding activity is higher in ravine CGIs. Boxplots show the distribution of POLR2A scores in CGI on a log scale. Violin plots show the density of the scores.

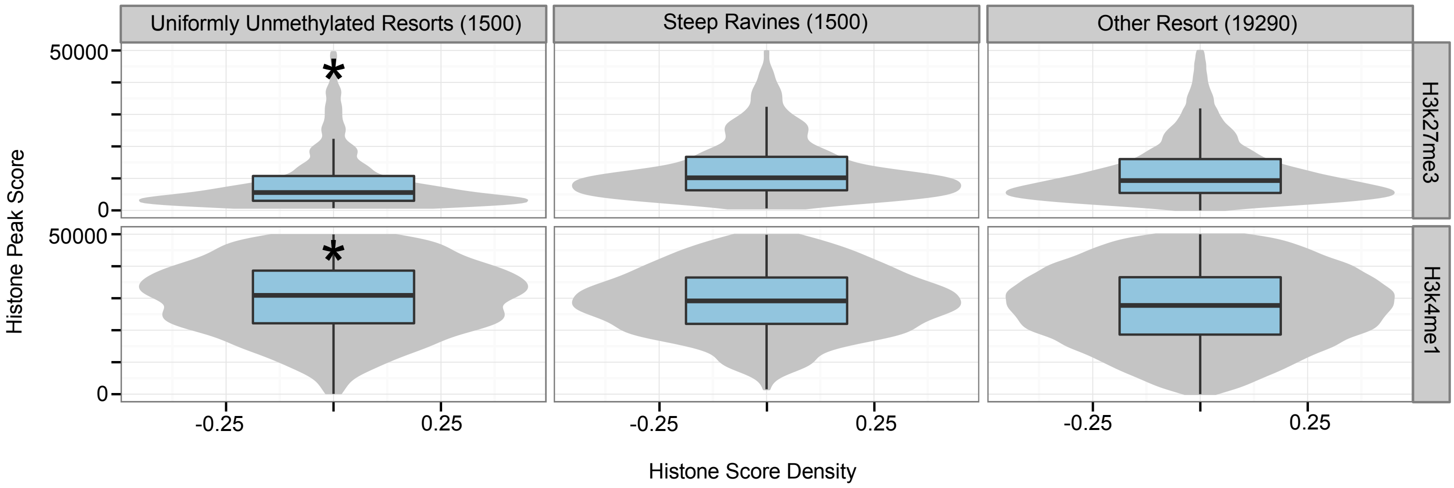

Supplementary Figure S11. Uniformly unmethylated resort CGIs show di erences in histone mark. Only data from 2 of the 12 ENCODE histone marks are shown (H3K27me3 and H3K4me1) as they are the only marks showing any significant differences between resort classes. Histone peak scores >50 000 (max in score data 165 000) are not shown so the di erences between resort classes are noticeable on the scale. Grey violin plots show the density of histone scores in each resort class.

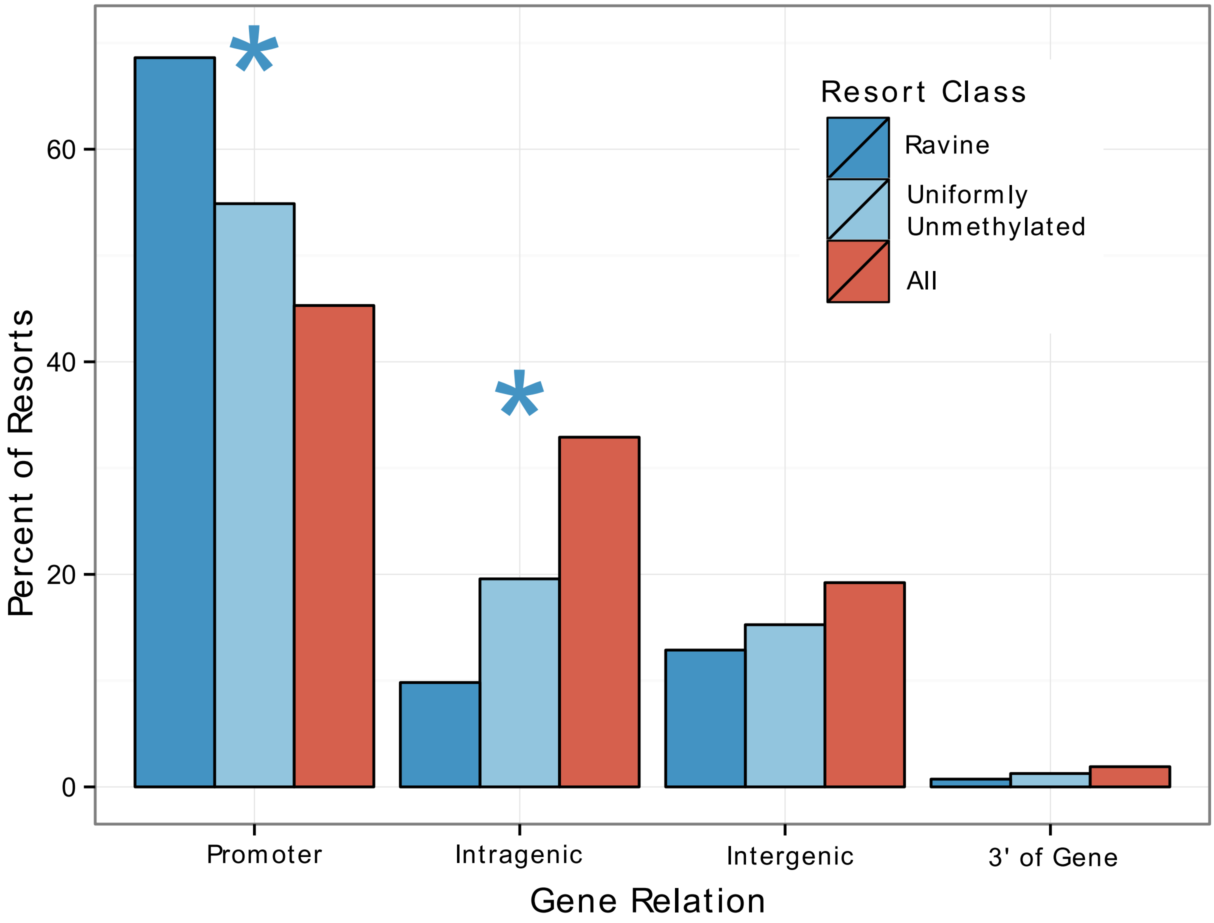

Supplementary Figure S12. Ravines are more frequent in promoters than other resorts. Bars show the percent of gene-associated CGIs located in either promoter, intragenic, 3' of gene, or intergenic. Asterisks indicate gene associations where ravines were significantly different.

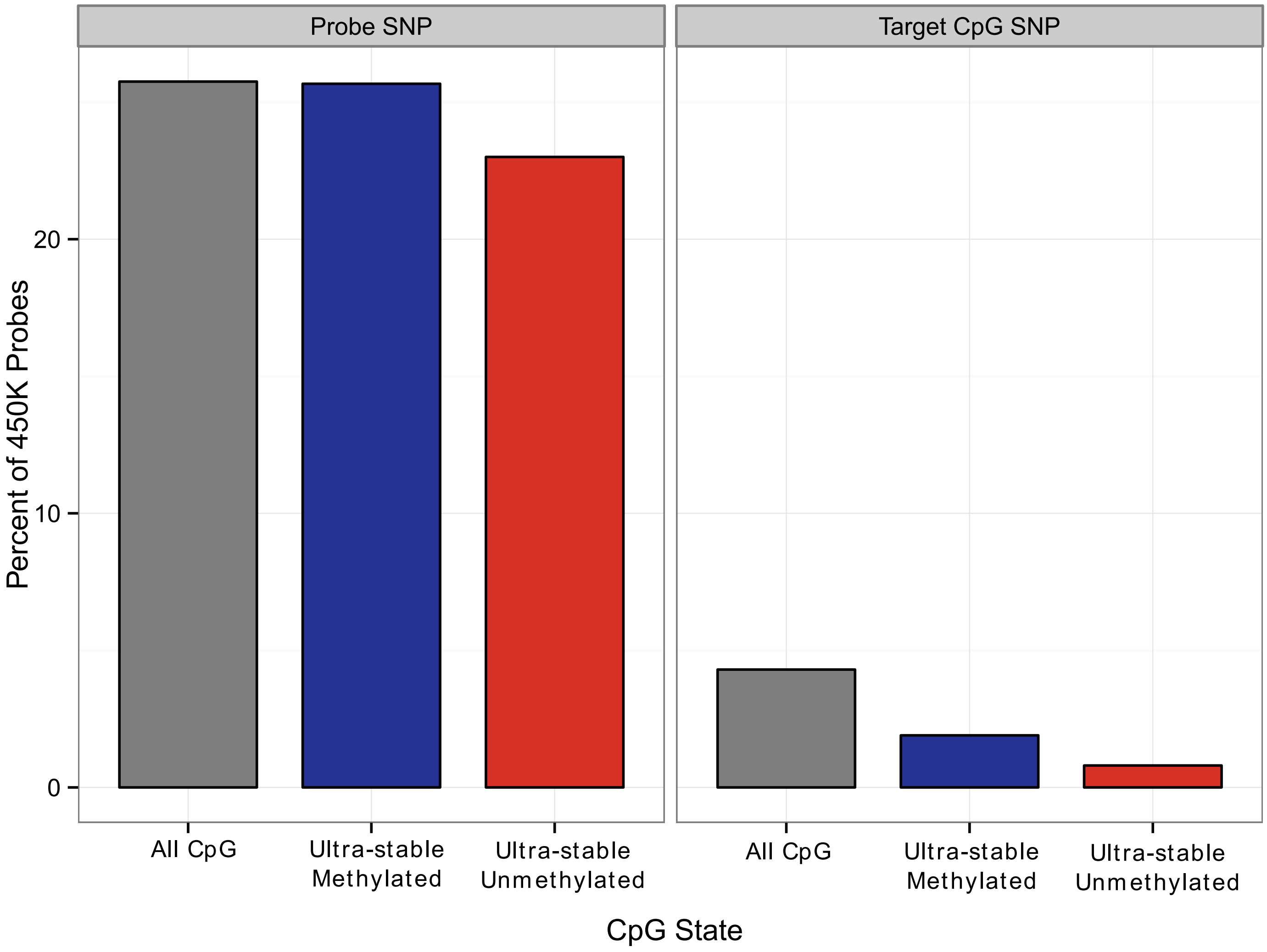

Supplementary Figure S13. Ultra-stable CpGs are not the result of SNPs affecting probe hybridization. Left panel shows the percent of 450K probes with a SNP in the probe body. Right panel shows the percent of probes with a SNP at the assayed CpG.

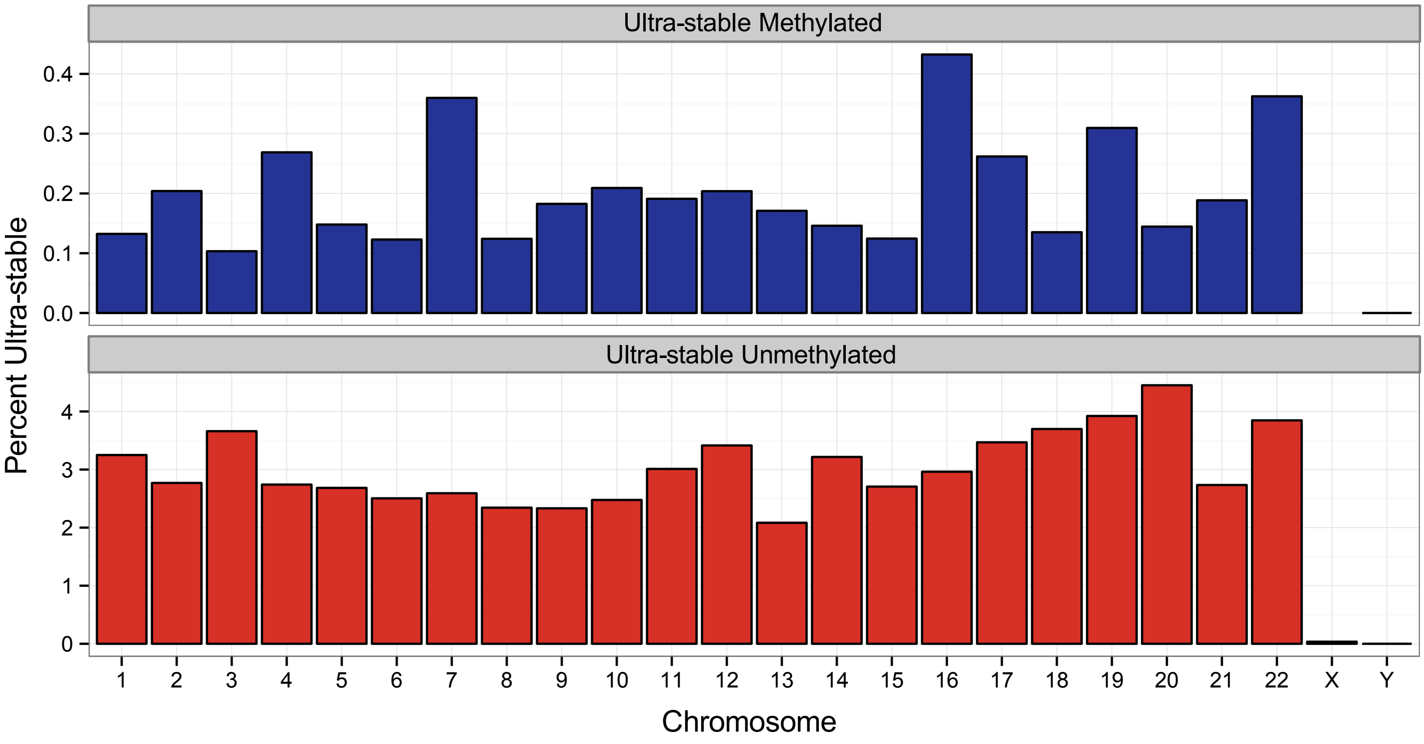

Supplementary Figure S14. Ultra-stable CpGs are distributed evenly across chromosomes. Percent of ultra-stable CpGs out of all probes on each chromosome is shown.

Table S1. Summary of the GEO series available on the 450K as of April 30, 2013. QC is the reason a series was not included in the analysis. Samples removed is the reason individual samples from a study were not included. Probes is the number of probes (of 485577) provided on GEO.

Available (April 30)	Samples	QC	Samples Used	Samples Removed	Probes	Data Type	Tissues	Pubmed ID
GSE38271	72	FFPE	0	-	485577	Beta	Head and neck	23419152
GSE43976	95	Filtered	0	-	473097	Beta	Blood	23422812
GSE45529	22	Filtered	0	-	441946	Beta	Blood, placenta, sperm, buccal	23538714
GSE41826	145	Filtered	0	-	480492	Beta	Brain	23426267
GSE42700	53	Filtered	0	-	330168	Beta	Buccal	NA
GSE42409	12	Filtered	0	-	428216	Beta	Buccal, blood, chorionic villus	23452981
GSE34486	16	Filtered	0	-	485512	Beta	Endothelium	22434260
GSE34487	16	Filtered	0	-	485512	Beta	Endothelium	22434260
GSE36278	142	Filtered	0	-	485512	Beta	Glioblastoma	NA
GSE37067	38	Filtered	0	-	350447	Beta	iPSC	NA
GSE37066	24	Filtered	0	-	350447	Beta	iPSC	NA
GSE34688	16	Filtered	0	-	350447	Beta	iPSC, ES	23032973
GSE46364	11	Filtered	0	-	485512	Beta	Joints (synoviocytes)	22736089
GSE37633	2	Filtered	0	-	483823	Beta	Liver cell line	NA
GSE40279	656	Filtered	0	-	473034	Beta	Whole Blood	NA
GSE30338	134	M Values	0	-	485577	Beta	glioma cells	
GSE40870	46	Methlytransferase	0	-	485577	Beta	Bone marrow	NA
GSE43851	20	Methlytransferase	0	-	485577	Beta	Cell line	23408854
GSE32286	139	Methlytransferase	0	-	485577	Beta	glioblastomas	NA
GSE44830	26	Methlytransferase	0	-	485577	Beta	Xenograft	
GSE20945	9	Multiple arrays	0	-	485577	Beta	Leukemia cell line	22439938
GSE42882	23	NA Count	0	-	485577	Beta	Nervous system	
GSE34639	48	Not Beta	0	-	462176	Signal intensity	Blood	NA
GSE43414	696	Not Beta	0	-	-	None	Brain	23631413
GSE40699	62	Not Beta	0	-	-	IDAT	Encode Tissues	NA
GSE34779	24	Not Beta	0	-	98090	M-Values	lymphoblast	NA
GSE34777	8	Not Beta	0	-	98090	M-Values	Lymphoblasts	NA
GSE41273	62	Low Sample Correlation	0	-	485577	Beta	Blood	23356558
GSE32283	86	Subseries	0	-	485577	Beta	Glioblastoma	NA
GSE38266	42	Subseries	0	-	485577	Beta	Head and neck	23419152
GSE38128	13	Superseries	0	-	485577	Beta	Blood	22919074
GSE42119	61	Superseries	0	-	485577	Beta	Bone marrow	23152544
GSE40871	115	Superseries	0	-	485577	Beta	Bone marrow	23297133

GSE32149	48	Superseries	0	-	485577	Beta	Colon mucosa, blood	NA
GSE33130	4	Superseries	0	-	485577	Beta	embryocarcinoma	22569366
GSE32079	68	Superseries	0	-	485577	Beta	Epithelial	NA
GSE40927	50	Superseries	0	-	485577	Beta	Fibroblasts	23202434
GSE30339	134	Superseries	0	-	485577	Beta	glioma	22343889
GSE30654	153	Superseries	0	-	485577	Beta	iPS,ES	22560082
GSE41117	132	Superseries	0	-	485577	Beta	oral squamous	22466171
GSE34403	8	Superseries	0	-	485577	Beta	Prostate	22466171
GSE45459	22	Used	18	NA Count	485577	Beta	B-cell	23074194
GSE46168	14	Used	13	NA Count	485577	Beta	B-cell	NA
GSE42865	16	Used	16	-	485577	Beta	B-cells	23257959
GSE40005	24	Used	23	NA Count	485577	Beta	blood	NA
GSE37966	7	Used	7	-	485577	Beta	blood, sperm	22919074
GSE42118	10	Used	10	-	485577	Beta	Bone marrow	NA
GSE39141	33	Used	33	-	485577	Beta	Bone marrow, b cell	23110451
GSE29290	22	Used	22	-	485577	Beta	Breast and colon	NA
GSE41114	46	Used	46	-	485577	Beta	Buccal, and blood	NA
GSE42752	63	Used	63	-	485577	Beta	colon	NA
GSE32362	4	Used	4	-	485577	Beta	embryocarcinoma	22569366
GSE40909	25	Used	25	-	485577	Beta	endothelial, stem	NA
GSE45198	28	Used	28	-	485577	Beta	glioma cells	23558169
GSE31848	153	Used	153	-	485577	Beta	hPSCs	22560082
GSE40790	4	Used	4	-	485577	Beta	ips, hsc	23386128
GSE36216	75	Used	75	-	485577	Beta	lung	22261801
GSE32148	48	Used	47	NA Count	485577	Beta	peripheral blood	22467598
GSE33233	19	Used	18	NA Count	485577	Beta	peripheral blood	22689993
GSE36064	78	Used	78	-	485577	Beta	peripheral blood	22300631
GSE42861	689	Used	689	-	485577	Beta	peripheral blood	23334450
GSE34340	6	Used	6	-	485577	Beta	prostate	NA
GSE38240	12	Used	12	-	485577	Beta	prostate	23345608
GSE43141	2	Used	2	-	485577	Beta	skin fibroblast	NA
GSE38268	6	Used	6	-	485577	Beta	tongue	NA
GSE38270	24	Used	24	-	485577	Beta	tongue	NA
GSE41336	90	Used	90	-	485577	Beta	trophoblast	23314690
GSE30870	40	Used	40	-	485577	Beta	whole blood	22689993
GSE37965	30	Used	30	-	485577	Beta	whole blood	23054610
GSE41169	95	Used	95	-	485577	Beta	whole blood	23034122
GSE35069	60	Used	60	-	485577	Beta	whole, peripheral blood	22848472
	**4614**	**30 series used**	**1737**					

Table S2. Summary of tissue types used in analysis.

**Tissue**	**Samples**
Adipose	2
Bladder	2
Blood	119
Blood (B Cell)	35
Blood (Peripheral)	841
Blood (Whole)	149
Blood Vessel	8
Bone Marrow	38
Brain	34
Breast	16
Colon	69
Epithelial	5
Fibroblast	9
Kidney	12
Liver	4
Lung	82
Lymph	2
Muscle	6
Oral	76
Pancreas	2
Prostate	18
Somatic Cell Line	21
Sperm	2
Spleen	5
Stem Cell	78
Stomach	6
Testes	4
Thymus	2
Trophoblast	90
**Total**	**1737**

Table S3. Ultra-stable CpGs loci. Probe_ID is the 450K CpG probe ID given by Illumina. State is the state of ultra-stable CpG. Coordinate_37 is the Human Genome Build 37 position. Chromosome_37 is the Human Genome Build 37 chromosome. See Additional file 2.

Table S4. Summary of each resort class overlap with previously reported methylome features. Percent of resorts overlapped by a previously defined domain are shown. Number of domains used for each domain type is shown in brackets. Canyons LMR and UMR are the number of mouse genome (mm9) regions successfully converted to human genome (hg19) coordinates.

	**TDMR/CDMR (12059/1866 regions)**	**RDMR (4401 regions)**	**Canyons (1092 domains)**	**cUMR (11964 regions)**	**Valleys (2380 Domains)**	**LMR (26335 regions)**	**UMR (33675 regions)**	**LRES (47 domains)**	**LREA (35 domains)**
**Uniformly Unmethylated (1500)**	1.73	1.93	25.12	60.33	36.87	0.20	42.69	4.68	2.08
**Ravine (1500)**	0.10	0.08	1.93	61.32	3.60	0.20	37.07	2.87	0.64
**Other (19290)**	18.53	12.47	3.03	70.8	5.78	0.24	27.20	2.74	0.96
	Irizarry *et al,.* 2009	Doi *et al.*, 2009	Jeong *et al.*, 2014		Xie *et al.* 2013	Stadler *et al,.* 2011		Coolen *et al.*, 2010	Bert *et al.*, 2013

Table S5. Summary of the GEO series available on the 450K between April 30, 2013 and July 29, 2013. QC is the reason a series was not included in the confirmation dataset. Probes is the number of probes (of 485577) provided on GEO.

**Available** (April 30-July 29)	**Samples**	**QC**	**Samples Used**	**Probes**	**Data Type**	**Tissues**	**Pubmed ID**
GSE41782	32	Filtered	0	137797	Beta	Blood	24039605
GSE44132	55	Filtered	0	483266	Beta	Blood	23689534
GSE44667	40	Filtered	0	430685	Beta	Placenta	23770704
GSE44684	67	Filtered	0	485512	Beta	Cerebellum	23660940
GSE46573	22	Filtered	0	441946	Beta	Blood, sperm, placenta, pancreas, buccal	23538714
GSE43226	12	Mouse samples	0	485577	Beta	Mouse (Bone marrow, liver, heart, spleen)	23639479
GSE42707	1	Sample size	0	485577	Beta	Embryonic stem cell	23598999
GSE42310	14	Superseries	0	485577	Beta	Colon	23598999
GSE43298	176	Superseries	0	485577	Beta	paraganglioma	23707781
GSE44712	56	Superseries	0	430685	Beta	placental	23770704
GSE44838	52	Superseries	0	485577	Beta	breast	23844228
GSE47639	5	Superseries	0	485577	Beta	esophageal	24022190
GSE36369	322	Used	255	485577	Beta	Cell line and blood	NA
GSE38235	92	Used	92	485577	Beta	Blood (B cell)	23722552
GSE39560	34	Used	34	485577	Beta	Saliva	23706164
GSE39672	133	Used	133	485577	Beta	Lymphoblastoid	23792949
GSE40853	51	Used	51	485577	Beta	Cartilage	23863747
GSE42308	6	Used	6	485577	Beta	Colon	23598999
GSE43293	24	Used	24	485577	Beta	Adrenal glands	23707781
GSE44798	50	Used	50	485577	Beta	Blood	23716672
GSE44837	26	Used	26	485577	Beta	Breast	23844228
GSE45707	4	Used	4	485577	Beta	Monocytes	NA
GSE45958	24	Used	24	485577	Beta	Breast	NA
GSE46650	12	Used	12	485577	Beta	Fibroblast	23306098
GSE47627	28	Used	28	485577	Beta	Sperm	23071498
GSE47637	2	Used	2	485577	Beta	Esophageal	24022190
GSE45199	16	Used	16	485577	Beta	Oligodendroglioma	23558169
	**1356**	**15 series used**	**757**				

Table S6. Summary of the resort classes features. 450K density represents the probes per base pair in a feature. Genomic CpG density represents the number of CpG per base pair present whether or not they are measured by the 450K. Mean methylation is the mean beta of all probes in a feature across 1737 samples. Methylation variability is the standard deviation of probe beta values across 1737 samples in a feature.

Resort Feature	Resort Class	450K Density	Genomic CpG Density	Mean Methylation	Methylation Variability
CGI	Uniformly Unmethylated	0.009	0.098	0.146	1.023e-03
	Ravine	0.009	0.107	0.081	6.465e-04
	Other	0.009	0.099	0.250	8.599e-04
Shores	Uniformly Unmethylated	0.003	0.025	0.187	1.486e-03
	Ravine	0.002	0.020	0.641	3.675e-03
	Other	0.002	0.021	0.465	1.088e-03
Shelves	Uniformly Unmethylated	0.001	0.018	0.438	5.060e-03
	Ravine	0.002	0.015	0.798	1.676e-03
	Other	0.001	0.017	0.717	8.477e-04

Table S7. Significantly over-represented (corrected p value <0.001) GO groups in the uniformly unmethylated resort associated genes. Columns are: name of the GO gene set, GO ID, number of genes in the GO gene set, original p value and Benjamini-Hochberg corrected p value (which the data is sorted by). Multifunctionality (MF) scores p values and corrected p values are provided, but corrected p value without MF correction is used for significance calling.

Name	ID	Number of Genes	p value	Corrected p value	MF p value	Corrected MF p value	MF
spinal cord development	GO:0021510	73	1.11E-17	5.278E-14	1.053E-07	1.669E-04	0.838
neuron fate commitment	GO:0048663	56	9.085E-16	2.16E-12	5.745E-08	2.732E-04	0.847
neuron migration	GO:0001764	94	2.722E-14	4.315E-11	1.297E-06	1.233E-03	0.93
outflow tract morphogenesis	GO:0003151	50	1.113E-12	1.323E-09	0.55269379	1	0.881
diencephalon development	GO:0021536	68	1.46E-12	1.388E-09	5.413E-06	3.678E-03	0.877
cell differentiation in spinal cord	GO:0021515	41	2.221E-12	1.76E-09	1.342E-05	7.091E-03	0.756
pallium development	GO:0021543	91	4.282E-12	2.909E-09	3.399E-06	2.694E-03	0.879
metanephros development	GO:0001656	80	9.006E-12	5.354E-09	0.08730415	1	0.886
forebrain generation of neurons	GO:0021872	51	1.827E-11	7.901E-09	1.194E-04	0.03155777	0.865
embryonic digestive tract development	GO:0048566	35	1.753E-11	8.338E-09	0.13851477	1	0.855
forebrain neuron differentiation	GO:0021879	40	1.734E-11	9.163E-09	6.427E-05	0.02183443	0.842
embryonic skeletal system morphogenesis	GO:0048704	84	2.858E-11	1.133E-08	2.798E-04	0.06048596	0.953
limbic system development	GO:0021761	71	3.247E-11	1.188E-08	6.635E-08	1.578E-04	0.866
ventral spinal cord development	GO:0021517	33	8.395E-11	2.852E-08	4.362E-04	0.07979735	0.758
embryonic digestive tract morphogenesis	GO:0048557	20	9.724E-11	3.083E-08	0.31962669	1	0.832
palate development	GO:0060021	69	1.329E-10	3.951E-08	5.028E-04	0.08539711	0.861
regulation of neural precursor cell proliferation	GO:2000177	47	3.656E-10	1.023E-07	0.01686011	0.95460318	0.86
forelimb morphogenesis	GO:0035136	37	6.101E-10	1.612E-07	0.01770803	0.95703859	0.745
cell fate specification	GO:0001708	61	6.477E-10	1.621E-07	3.357E-05	0.01451621	0.853
peripheral nervous system neuron differentiation	GO:0048934	12	1.217E-09	2.631E-07	3.463E-04	0.06863086	0.451
peripheral nervous system neuron development	GO:0048935	12	1.217E-09	2.631E-07	3.463E-04	0.06863086	0.451
positive regulation of neuron differentiation	GO:0045666	63	1.199E-09	2.715E-07	8.234E-04	0.11188962	0.837
cardiac chamber morphogenesis	GO:0003206	99	1.182E-09	2.81E-07	0.50484358	1	0.887
cardiac septum development	GO:0003279	57	1.422E-09	2.941E-07	0.64227964	1	0.872
embryonic forelimb morphogenesis	GO:0035115	29	1.925E-09	3.816E-07	5.334E-03	0.46981081	0.723
forebrain regionalization	GO:0021871	20	2.173E-09	4.133E-07	7.995E-05	0.02376578	0.697
neuron fate specification	GO:0048665	25	3.071E-09	5.617E-07	5.117E-05	0.02028098	0.709
cardiac ventricle development	GO:0003231	91	6.112E-09	1.002E-06	0.60352184	1	0.885
central nervous system neuron development	GO:0021954	55	5.887E-09	1.037E-06	5.241E-05	0.01917466	0.853

ureteric bud development	GO:0001657	91	6.112E-09	1.038E-06	0.26643212	1	0.885
midbrain development	GO:0030901	32	8.387E-09	1.33E-06	8.822E-07	1.049E-03	0.696
regulation of glial cell differentiation	GO:0045685	44	1.025E-08	1.524E-06	0.01190884	0.77586918	0.863
hindlimb morphogenesis	GO:0035137	38	1.004E-08	1.541E-06	0.02003856	1	0.81
embryonic digit morphogenesis	GO:0042733	46	2.048E-08	2.951E-06	4.24E-03	0.42006388	0.818
dorsal/ventral pattern formation	GO:0009953	89	2.142E-08	2.997E-06	1.508E-04	0.03775389	0.972
cerebral cortex development	GO:0021987	60	2.544E-08	3.362E-06	5.361E-04	0.08791468	0.86
regulation of gliogenesis	GO:0014013	60	2.544E-08	3.458E-06	0.02061329	1	0.877
embryonic camera-type eye development	GO:0031076	35	3.034E-08	3.9E-06	0.04776796	1	0.846
mesenchymal cell development	GO:0014031	99	3.206E-08	4.012E-06	0.67746894	1	0.886
dorsal spinal cord development	GO:0021516	20	4.007E-08	4.886E-06	7.995E-05	0.02535017	0.517
spinal cord motor neuron differentiation	GO:0021522	25	4.419E-08	5.254E-06	2.419E-03	0.26750416	0.665
cerebral cortex neuron differentiation	GO:0021895	16	5.132E-08	5.953E-06	1.445E-05	6.872E-03	0.76
negative regulation of neuron apoptotic process	GO:0043524	94	5.953E-08	6.741E-06	0.01855004	0.99128066	0.882
negative regulation of neuron death	GO:1901215	94	5.953E-08	6.741E-06	0.01855004	0.99128066	0.882
cardiac septum morphogenesis	GO:0060411	43	6.217E-08	6.877E-06	0.7150241	1	0.867
embryonic camera-type eye morphogenesis	GO:0048596	26	7.263E-08	7.851E-06	0.05877479	1	0.828
camera-type eye morphogenesis	GO:0048593	90	1.345E-07	1.422E-05	0.0330986	1	0.878
ventricular septum development	GO:0003281	40	1.891E-07	1.955E-05	0.67687288	1	0.848
negative regulation of neurogenesis	GO:0050768	92	1.975E-07	1.999E-05	0.03771388	1	0.984
regulation of oligodendrocyte differentiation	GO:0048713	23	2.118E-07	2.098E-05	0.03981093	1	0.791
digestive tract morphogenesis	GO:0048546	54	2.247E-07	2.181E-05	0.6054949	1	0.883
pancreas development	GO:0031016	69	2.403E-07	2.286E-05	0.09958755	1	0.865
face morphogenesis	GO:0060325	29	2.774E-07	2.537E-05	0.08173651	1	0.788
embryonic hindlimb morphogenesis	GO:0035116	29	2.774E-07	2.586E-05	0.02311755	1	0.796
pituitary gland development	GO:0021983	42	3.602E-07	3.232E-05	0.03142116	1	0.856
ureteric bud morphogenesis	GO:0060675	56	3.788E-07	3.336E-05	0.40139621	1	0.872
hippocampus development	GO:0021766	49	3.877E-07	3.353E-05	8.117E-05	0.02270861	0.837
lung morphogenesis	GO:0060425	36	4.106E-07	3.487E-05	0.01557035	0.91422918	0.848
regulation of stem cell proliferation	GO:0072091	72	4.642E-07	3.873E-05	7.753E-03	0.58532528	0.881
head development	GO:0060322	50	5.103E-07	4.185E-05	0.065048	1	0.835
cardiac chamber formation	GO:0003207	11	5.375E-07	4.333E-05	0.47784594	1	0.735
face development	GO:0060324	37	5.761E-07	4.567E-05	0.05846942	1	0.809
embryonic eye morphogenesis	GO:0048048	31	6.105E-07	4.76E-05	0.09914553	1	0.836
stem cell proliferation	GO:0072089	52	8.644E-07	6.423E-05	0.07583075	1	0.856
proximal/distal pattern formation	GO:0009954	32	8.822E-07	6.455E-05	7.438E-06	4.422E-03	0.736
metencephalon development	GO:0022037	75	8.631E-07	6.516E-05	1.087E-03	0.14358641	0.859
odontogenesis	GO:0042476	92	9.18E-07	6.516E-05	0.15717717	1	0.984
bone morphogenesis	GO:0060349	67	8.542E-07	6.552E-05	0.03714177	1	0.859

inner ear morphogenesis	GO:0042472	92	9.18E-07	6.615E-05	7.26E-04	0.10462652	0.965
cardiac right ventricle morphogenesis	GO:0003215	16	9.678E-07	6.769E-05	0.61143545	1	0.747
endocrine pancreas development	GO:0031018	46	1.159E-06	7.991E-05	0.01507255	0.89606301	0.825
head morphogenesis	GO:0060323	33	1.255E-06	8.29E-05	0.11811378	1	0.807
nephron development	GO:0072006	77	1.28E-06	8.34E-05	0.82565216	1	0.886
thymus development	GO:0048538	33	1.255E-06	8.407E-05	0.11811378	1	0.81
regulation of organ formation	GO:0003156	33	1.255E-06	8.527E-05	0.29321985	1	0.852
body morphogenesis	GO:0010171	40	1.481E-06	9.516E-05	0.07687415	1	0.817
embryonic heart tube development	GO:0035050	62	1.569E-06	9.817E-05	0.28226955	1	0.876
cardiac ventricle morphogenesis	GO:0003208	62	1.569E-06	9.948E-05	0.69805712	1	0.877
embryonic heart tube morphogenesis	GO:0003143	49	2.548E-06	1.534E-04	0.3088397	1	0.863
positive regulation of glial cell differentiation	GO:0045687	23	2.543E-06	1.551E-04	0.38321525	1	0.81
embryonic skeletal joint morphogenesis	GO:0060272	13	2.525E-06	1.559E-04	5.363E-04	0.08501935	0.663
positive regulation of neural precursor cell proliferation	GO:2000179	29	2.704E-06	1.587E-04	0.02311755	1	0.844
olfactory bulb development	GO:0021772	29	2.704E-06	1.607E-04	5.334E-03	0.47867516	0.806
regulation of embryonic development	GO:0045995	82	3.225E-06	1.87E-04	0.50993673	1	0.884
cardiac neural crest cell development involved in heart development	GO:0061308	6	3.509E-06	1.987E-04	1	1	0.678
noradrenergic neuron differentiation	GO:0003357	6	3.509E-06	2.011E-04	0.29839077	1	0.581
regulation of DNA binding	GO:0051101	66	3.647E-06	2.041E-04	0.73773033	1	0.869
olfactory lobe development	GO:0021988	30	3.848E-06	2.128E-04	6.351E-03	0.5119186	0.812
negative regulation of neuron differentiation	GO:0045665	51	4.152E-06	2.27E-04	0.02550863	1	0.816
cardiocyte differentiation	GO:0035051	84	4.563E-06	2.438E-04	0.71707901	1	0.886
heart looping	GO:0001947	44	4.518E-06	2.442E-04	0.2440901	1	0.859
negative regulation of neural precursor cell proliferation	GO:2000178	14	4.799E-06	2.481E-04	0.0425761	1	0.686
bone development	GO:0060348	93	4.7E-06	2.484E-04	0.16394622	1	0.987
aorta morphogenesis	GO:0035909	19	4.869E-06	2.49E-04	0.67458076	1	0.822
spinal cord association neuron differentiation	GO:0021527	14	4.799E-06	2.508E-04	7.95E-04	0.11120716	0.509
metanephric nephron development	GO:0072210	31	5.389E-06	2.727E-04	0.53759448	1	0.869
cerebral cortex cell migration	GO:0021795	25	5.729E-06	2.838E-04	3.852E-04	0.07328463	0.835
heart valve development	GO:0003170	25	5.729E-06	2.868E-04	0.77177601	1	0.848
cardiac ventricle formation	GO:0003211	10	6.025E-06	2.924E-04	0.44606208	1	0.712
forebrain cell migration	GO:0021885	38	5.989E-06	2.936E-04	2.329E-04	0.05538064	0.863
mesonephros development	GO:0001823	20	7.707E-06	3.702E-04	0.69326256	1	0.836
positive regulation of ossification	GO:0045778	39	7.912E-06	3.763E-04	0.90031753	1	0.859
determination of heart left/right asymmetry	GO:0061371	47	9.542E-06	4.449E-04	0.28264822	1	0.864
autonomic nervous system development	GO:0048483	47	9.542E-06	4.493E-04	0.75993499	1	0.855
endoderm development	GO:0007492	55	1.022E-05	4.542E-04	0.09378184	1	0.855

cell-cell signaling involved in cell fate commitment	GO:0045168	33	1.013E-05	4.544E-04	0.0383238	1	0.839
nephron epithelium development	GO:0072009	40	1.035E-05	4.558E-04	0.4046518	1	0.872
neural tube patterning	GO:0021532	33	1.013E-05	4.587E-04	0.0383238	1	0.81
positive regulation of gliogenesis	GO:0014015	33	1.013E-05	4.631E-04	0.29321985	1	0.841
neural crest cell differentiation	GO:0014033	63	1.012E-05	4.673E-04	0.70838321	1	0.879
cardiac neural crest cell differentiation involved in heart development	GO:0061307	7	1.17E-05	5.013E-04	1	1	0.721
aorta development	GO:0035904	21	1.183E-05	5.021E-04	0.7108729	1	0.835
hard palate development	GO:0060022	7	1.17E-05	5.059E-04	0.05690589	1	0.36
telencephalon regionalization	GO:0021978	7	1.17E-05	5.105E-04	5.527E-03	0.46939426	0.38
spinal cord motor neuron cell fate specification	GO:0021520	11	1.261E-05	5.307E-04	0.02193044	1	0.536
embryonic skeletal joint development	GO:0072498	16	1.445E-05	6.028E-04	1.576E-03	0.18734492	0.703
branching involved in ureteric bud morphogenesis	GO:0001658	49	1.515E-05	6.266E-04	0.3088397	1	0.86
embryonic cranial skeleton morphogenesis	GO:0048701	35	1.808E-05	7.351E-04	0.13851477	1	0.829
telencephalon cell migration	GO:0022029	35	1.808E-05	7.415E-04	6.678E-04	0.09925519	0.863
neural tube closure	GO:0001843	67	2.138E-05	8.618E-04	0.03714177	1	0.84
neural tube formation	GO:0001841	85	2.262E-05	9.042E-04	0.11375899	1	0.955
retinal ganglion cell axon guidance	GO:0031290	17	2.335E-05	9.254E-04	2.507E-04	0.05677822	0.567
forebrain neuron development	GO:0021884	12	2.399E-05	9.431E-04	3.67E-03	0.38789957	0.597
negative regulation of glial cell differentiation	GO:0045686	23	2.571E-05	9.86E-04	8.693E-03	0.60798514	0.794
lens morphogenesis in camera-type eye	GO:0002089	23	2.571E-05	9.94E-04	0.03981093	1	0.691
tube closure	GO:0060606	68	2.552E-05	9.95E-04	0.04006723	1	0.843

Table S8. Significantly over-represented (corrected p value <0.001) DO groups in the uniformly unmethylated resort associated genes. Columns are: name of the DO gene set, DO ID, number of genes in the DO gene set, original p value and Benjamini-Hochberg corrected p value (which the data is sorted by). Multifunctionality (MF) scores p values and corrected p values are provided, but corrected p value without MF correction is used for significance calling.

Name	ID	Number of Genes	p value	Corrected p value	MF p value	Corrected MF p value	MF
intellectual disability	DOID_1059	524	1.292E-09	1.979E-06	1.586E-03	1	0.924
autistic disorder	DOID_12849	299	2.632E-09	2.016E-06	7.415E-03	1	0.923
pervasive developmental disorder	DOID_0060040	805	1.441E-08	7.357E-06	0.01077664	1	0.893
physical disorder	DOID_0080015	264	5.064E-08	1.939E-05	2.598E-03	0.99503206	0.922
autism spectrum disorder	DOID_0060041	798	1.718E-07	4.387E-05	0.0266294	1	0.892
